# Global burden and future trends of gastric cancer in women of reproductive age: estimates from the GBD 2021 Study, 1990–2050

**DOI:** 10.3389/fonc.2025.1616936

**Published:** 2025-11-14

**Authors:** Nan Jiang

**Affiliations:** Department of Gastroenterology, Dahua Hospital, Xuhui District, Shanghai, China

**Keywords:** gastric cancer, global burden of disease, women of reproductive age, epidemiology, socio-demographic index, disability-adjusted life years, disease trends, forecasting

## Abstract

**Background:**

Gastric cancer (GC) is the fifth most common malignancy and the third leading cause of cancer-related mortality worldwide. Although incidence is higher in men, GC remains a significant health issue for women of reproductive age (15–49 years) due to biological, hormonal, and socioeconomic factors. However, this population has been underrepresented in cancer surveillance. This study assessed global, regional, and national GC burden among women from 1990 to 2021 and projected future trends to 2050 using Global Burden of Disease (GBD) 2021 data.

**Methods:**

We analyzed GBD 2021 data from 204 countries and territories for females aged 15–49. Indicators included incidence, mortality, disability-adjusted life years (DALYs), and age-standardized rates (ASRs). Temporal trends were quantified using estimated annual percentage change (EAPC) and Joinpoint regression, stratified by Socio-demographic Index (SDI). Forecasts to 2050 were derived from age-period-cohort modeling. This study follows GATHER guidelines.

**Results:**

From 1990 to 2021, the GC burden in women aged 15–49 declined globally: ASPR decreased from 10.42 to 5.41 per 100,000 (EAPC −2.11), ASIR from 4.61 to 2.13 (EAPC −2.56), ASMR from 2.02 to 0.89 (EAPC −3.02), and ASDR from 59.6 to 23.8 per 100,000 (EAPC −3.00). High-SDI regions achieved the steepest reductions, while low-SDI regions showed modest progress. The burden peaked in women aged 45–49. By 2050, ASIR is projected to reach 2.81 per 100,000 persons (95% CI: 2.06, 3.56), reflecting an increase relative to 2021, largely in low- and middle-SDI countries.

**Conclusions:**

Despite overall global declines, substantial regional and socioeconomic disparities persist. High-SDI regions benefit from improved healthcare and screening, whereas low-SDI regions face slower progress. Strengthening H. pylori screening, early detection, dietary interventions, and healthcare equity is essential. Region-specific strategies are needed to further reduce the GC burden among women of reproductive age.

## Introduction

Gastric cancer (GC) remains one of the leading causes of cancer-related morbidity and mortality worldwide, with substantial variations observed by region, socioeconomic factors, and demographic characteristics. It is the fifth most common cancer and the third leading cause of cancer death globally (1). According to the Global Burden of Disease (GBD) Study, GC accounted for nearly 1,000,000 deaths in 2021, 329,823 of whom were women,29057 of whom aged15-49 (2).

In terms of sex-specific epidemiology, gastric cancer has a significantly higher incidence in men compared to women, with a reported male-to-female ratio of approximately 2:1 (3). However, this disparity is not universal and can vary by geographical region. In regions such as East Asia and Eastern Europe, the gender gap is narrower, and in some cases, women show a higher proportion of GC cases. Female patients tend to be younger at diagnosis and exhibit lower 5-year overall survival compared to males (4). In women of reproductive age, gastric cancer can have a profound impact because of its potential to interfere with fertility and maternal health. Although the incidence of GC in this age group is lower than in older women, studies have shown that younger patients are more likely to present with high-grade cancers—such as Borrmann type IV, signet ring cell carcinoma, and diffuse-type gastric cancers—and to experience more aggressive tumor behavior and poorer survival outcomes (5, 6). Furthermore, certain risk factors for GC, such as Helicobacter pylori infection, may have distinct effects on women’s health during their reproductive years, leading to adverse pregnancy outcomes—for example, an increased risk of hypertensive disorders (7). These factors, compounded by a lack of early screening and awareness, exacerbate the burden on women in this age group.

Moreover, the socioeconomic burden of gastric cancer is substantially higher in regions with limited healthcare infrastructure, where both early detection and effective treatments are often inadequate or unavailable (8). In low- and middle-income countries (LMICs), where gastric cancer is more prevalent, women of reproductive age often face a dual burden: cancer-related morbidity and mortality exacerbated by gender-based disparities in healthcare access.

Gastric cancer remains a major public health concern, particularly among women of reproductive age. While there has been progress in the realm of early detection and treatment, the research landscape concerning gastric cancer in this demographic has not been comprehensively investigated at global, regional, and national levels. Furthermore, the ability to forecast disease trends is paramount for the effective implementation of prevention and control strategies.

This study aims to analyze the global, regional, and national trends in the burden of gastric cancer among women aged 15–49 from 1990 to 2021, using data from the 2021 Global Burden of Disease (GBD) study. In addition, it projects the future burden through 2050 to provide a forward-looking perspective on disease dynamics. By examining incidence, prevalence, DALYs, and mortality, we sought to identify epidemiological patterns, disparities across different SDI levels, and potential future trends. The findings will provide valuable insights for policymakers and healthcare professionals to optimize prevention, screening, and treatment strategies for gastric cancer in women of reproductive age.

## Methods

### Sources of information

This study utilized data from the Global Burden of Disease (GBD) 2021 database, and produced comprehensive estimates if stomach cancer in females of reproductive age according to age, location in 204 countries and territories from 1990-2021.

We extracted information on stomach cancer burden specifically among women of reproductive age (15–49 years) from 1990 to 2021, focusing on key epidemiological indicators, including: incidence, Mortality, Disability-Adjusted Life Years (DALYs), Age-Standardized Rates (ASR) per 100,000 population, Socio-Demographic Index (SDI) stratification.

All estimates were derived from GBD modeling methods, which integrate multiple data sources such as cancer registries, hospital records, vital statistics, and systematic literature reviews. Data processing and analysis were performed using the GBD Results Tool provided by IHME.

The study adheres to the Guidelines for Accurate and Transparent Health Estimates Reporting (GATHER) (9) to ensure the reliability and validity of the findings.

### Reference for data source

Global Burden of Disease Collaborative Network. Global Burden of Disease Study 2021 (GBD 2021) Results. Seattle, United States: Institute for Health Metrics and Evaluation (IHME), 2022. Available from https://vizhub.healthdata.org/gbd-results/.

### Indicators analysed

The following indicators were utilised in this study to describe the global burden of disease for gastric cancer in women of reproductive age: incidence, prevalence, estimates of DALYs (disability-adjusted life years) and mortality rates, and their 95% uncertainty intervals (UIs). The study utilized the age structure of the GBD world standard population to calculate age-standardized incidence rate (ASIR), age-standardized prevalence rate (ASPR), age-standardized DALY rate (ASDR), age-standardized mortality rate (ASMR).

### Statistical methods

A comprehensive analysis was carried out on global data regarding the disease burden of gastric cancer in women of reproductive age from 1990 to 2021. The study employed Excel 2019 and R (version 4.3.3) for data processing and statistical evaluation. Data underwent cleaning, organization, and subsequent analysis, with visualization performed using R software’s dplyr, officer, and ggplot2 packages. Statistical significance was defined as a p-value below 0.05.

#### Trend Analyses

##### Percentage change (EAPC)

The variation in disease burden by age, sex, region, and country from 1990 to 2021 was computed and visualized using R software. Trends in age-standardized rates were assessed through Estimated Annual Percentage Change (EAPC), where an EAPC with a 95% confidence interval lower bound exceeding 0 indicated an increasing trend, while a value below 0 suggested a decline (10, 11).

EAPC was applied to quantify overall temporal trends across the study period, while Joinpoint regression was used to identify potential inflection points and assess changes in trends across sub-periods. These complementary methods allowed for a more robust evaluation of temporal dynamics.

### Joinpoint regression model

To examine temporal trends from 1990 to 2021, the Joinpoint regression model was applied, estimating both the Average Annual Percent Change (AAPC) and the Annual Percent Change (APC) for different periods. Trends were considered stable if the 95% confidence interval included 0. AAPC or APC values significantly above or below 0 indicated upward or downward trends, respectively. The analysis was conducted using Joinpoint software (version 4.9.0.0), with results processed and visualized in R (12).

### SDI stratification

Countries and territories were stratified into five groups (low, low-middle, middle, high-middle, and high SDI) according to the socio-demographic index (SDI) used in the Global Burden of Disease 2021 framework. This allowed assessment of disparities in gastric cancer burden across different levels of socioeconomic development. Analyses were conducted at both national (204 countries/territories) and regional (21 GBD regions) levels.

### Forecasting and potential confounding

Forecasts extrapolate historical APC patterns and do not explicitly model exogenous shocks (e.g., COVID-19). To mitigate potential bias, we considered strategies described in the robustness section. We also note greater forecast uncertainty in low-SDI settings due to sparser data and rapidly changing health-system conditions.

### Robustness and uncertainty

While no formal sensitivity analyses were conducted due to constraints inherent to the GBD framework and data availability, robustness was enhanced through triangulation across multiple indicators (incidence, prevalence, mortality, and DALYs), two complementary trend estimators (EAPC and Joinpoint), and multi-level stratification (global, SDI, region, and country). All estimates are reported with 95% uncertainty intervals following GBD conventions.

### Pandemic-period data

This work retained 2020–2021 in the primary analyses because these years capture real-world disruptions in screening, diagnosis, and cancer care delivery, thereby preserving policy-relevant information on health-system performance under stress. We recognize that pandemic-related changes in ascertainment and service access may introduce uncertainty; therefore, trends spanning this period should be interpreted with caution, particularly in low-SDI settings.

## Results

### Global overview on the changes in the GBD of gastric cancer in women aged 15–49

From 1990 to 2021, the global burden of gastric cancer in women of reproductive age showed a consistent decline across all four metrics. The global age-standardized prevalence dropped from 10.42 (95% UI: 9.39–11.51) to 5.41 (4.78–6.18), with an EAPC of -2.11 (95% CI: -2.15, -2.07). Incidence also declined (EAPC: -2.56), decreasing from 4.61 (95% UI: 4.11–5.15) to 2.13 (95% UI: 1.89–2.41). Similarly, DALYs and deaths fell substantially, with EAPCs of -3.00 and -3.02 respectively, indicating improvements in survival and disease management over the past three decades (**Table 1**).

**Table 1:** Age-standardized stomach cancer burden results for the global female aged 15-49, five SDI regions, and 21 GBD regions.

Location		Prevalence			Incidence			DAYLs			Deaths		
Global	Location Name	EAPC (95%CI)	1990 (95%UI)	2021 (95%UI)	EAPC (95%CI)	1990 (95%UI)	2021 (95%UI)	EAPC (95%CI)	1990 (95%UI)	2021 (95%UI)	EAPC (95%CI)	1990 (95%UI)	2021 (95%UI)
	Global	-2.11 (-2.15, -2.07)	10.42 (9.39, 11.51)	5.41 (4.78, 6.18)	-2.56 (-2.60, -2.52)	4.61 (4.11, 5.15)	2.13 (1.89, 2.41)	-3.00 (-3.08, -2.93)	173.76 (153.39, 195.06)	71.45 (63.78, 79.53)	-3.02 (-3.09, -2.94)	3.48 (3.07, 3.91)	1.43 (1.28, 1.59)
SDI													
	High SDI	-2.22 (-2.32, -2.12)	14.75 (13.63, 15.87)	6.89 (6.24, 7.60)	-2.78 (-2.87, -2.69)	4.74 (4.41,5.06)	1.92 (1.77,2.11)	-3.53 (-3.63,-3.43)	127.54 (118.48,135.70)	42.03 (39.09,45.36)	-3.52 (-3.61,-3.42)	2.54 (2.37,2.69)	0.84 (0.78, 0.91)
	High-middle SDI	-1.67 (-1.77, -1.57)	12.61 (10.96, 14.46)	7.62 (6.19, 9.50)	-2.63 (-2.74, -2.52)	5.93 (5.14,6.83)	2.73 (2.25,3.36)	-3.68 (-3.84,-3.52)	227.24 (196.30,262.28)	77.68 (65.16,92.81)	-3.71 (-3.88,-3.55)	4.58 (3.96,5.28)	1.56 (1.31, 1.86)
	Middle SDI	-2.10 (-2.17, -2.03)	10.31 (8.50, 12.54)	5.52 (4.59, 6.71)	-2.83 (-2.90, -2.75)	5.17 (4.27,6.28)	2.24 (1.90,2.69)	-3.52 (-3.61,-3.43)	209.78 (174.14,254.15)	74.56 (64.45,87.15)	-3.53 (-3.62,-3.44)	4.21 (3.50,5.10)	1.49 (1.29, 1.75)
	Low-middle SDI	-1.25 (-1.29, -1.20)	5.03 (4.20, 5.82)	3.38 (2.92, 3.87)	-1.38 (-1.43, -1.34)	2.64 (2.21,3.05)	1.69 (1.46,1.94)	-1.56 (-1.61,-1.50)	116.53 (97.23,134.95)	71.09 (61.29,81.50)	-1.53 (-1.58,-1.48)	2.32 (1.94,2.69)	1.42 (1.23, 1.63)
	Low SDI	-1.50 (-1.57, -1.43)	5.85 (4.41, 7.13)	3.84 (3.02, 4.59)	-1.62 (-1.69, -1.54)	3.18 (2.40,3.86)	2.02 (1.59,2.41)	-1.67 (-1.74,-1.60)	141.59 (106.59,172.74)	88.50 (69.11,105.95)	-1.68 (-1.75,-1.60)	2.85 (2.15,3.47)	1.77 (1.39, 2.12)
GBD 21 regions													
	Andean Latin America	-0.98 (-1.17, -0.79)	12.69 (10.20, 15.67)	10.11 (7.36, 13.70)	-1.50 (-1.70,-1.31)	6.39 (5.16,7.90)	4.40 (3.25,5.89)	-1.88 (-2.07,-1.68)	276.75 (222.77,341.60)	169.83 (125.48,227.19)	-1.86 (-2.06,-1.67)	5.50 (4.44,6.78)	3.39 (2.51,4.54)
	Australasia	-0.87 (-1.01, -0.73)	4.78 (3.97, 5.77)	3.37 (2.69, 4.20)	-1.28 (-1.40,-1.16)	1.69 (1.42,2.02)	1.06 (0.86,1.29)	-1.75 (-1.86,-1.63)	46.82 (40.21,54.82)	25.41 (21.34,30.33)	-1.72 (-1.83,-1.60)	0.93 (0.80,1.09)	0.51 (0.43,0.61)
	Caribbean	-0.62 (-0.77, -0.48)	4.96 (4.05, 6.12)	3.99 (2.93, 5.38)	-0.77 (-0.92,-0.61)	2.49 (2.03,3.09)	1.95 (1.43,2.66)	-0.76 (-0.91,-0.61)	105.74 (84.60,132.49)	82.19 (58.82,112.80)	-0.80 (-0.96,-0.65)	2.10 (1.69,2.62)	1.62 (1.17,2.23)
	Central Asia	-2.88 (-2.98, -2.79)	10.53 (9.74, 11.39)	4.22 (3.58, 4.95)	-3.02 (-3.11,-2.92)	5.44 (5.03,5.88)	2.09 (1.78,2.44)	-3.17 (-3.26,-3.07)	235.98 (218.26,255.04)	87.44 (74.34,102.57)	-3.16 (-3.26,-3.07)	4.73 (4.37,5.12)	1.75 (1.49,2.05)
	Central Europe	-2.22 (-2.39, -2.04)	5.53 (5.17, 5.91)	2.77 (2.45, 3.10)	-2.59 (-2.75,-2.42)	2.73 (2.56,2.90)	1.24 (1.11,1.39)	-2.99 (-3.13,-2.85)	112.22 (105.20,119.33)	45.77 (40.78,50.85)	-2.96 (-3.11,-2.81)	2.26 (2.12,2.41)	0.93 (0.83,1.04)
	Central Latin America	-0.27 (-0.34, -0.19)	7.63 (7.18, 8.12)	7.04 (5.95, 8.18)	-0.78 (-0.87,-0.69)	3.66 (3.44,3.89)	2.93 (2.49,3.39)	-1.04 (-1.13,-0.95)	154.19 (145.38,163.86)	113.45 (96.46,130.99)	-1.06 (-1.15,-0.97)	3.09 (2.91,3.28)	2.27 (1.93,2.62)
	Central Sub-Saharan Africa	-1.18 (-1.25, -1.12)	4.20 (2.83, 6.01)	2.97 (1.94, 4.24)	-1.27 (-1.33,-1.22)	2.26 (1.53,3.23)	1.56 (1.02,2.23)	-1.34 (-1.40,-1.29)	100.07 (67.36,142.38)	67.48 (44.39,96.49)	-1.34 (-1.39,-1.28)	2.01 (1.36,2.86)	1.36 (0.90,1.94)
	Eastern Europe	-2.28 (-2.40, -2.15)	15.86 (15.12, 16.64)	8.09 (7.11, 9.09)	-2.84 (-2.96,-2.72)	6.95 (6.61,7.32)	2.99 (2.61,3.40)	-3.39 (-3.53,-3.24)	255.00 (242.09,269.01)	94.57 (82.00,109.33)	-3.41 (-3.55,-3.26)	5.17 (4.91,5.45)	1.90 (1.65,2.20)
	Eastern Sub-Saharan Africa	-2.35 (-2.52, -2.18)	5.92 (4.39, 7.62)	3.24 (2.52, 4.20)	-2.53 (-2.70,-2.35)	3.21 (2.38,4.13)	1.68 (1.31,2.18)	-2.61 (-2.78,-2.44)	143.05 (105.71,183.37)	72.94 (56.75,94.74)	-2.62 (-2.80,-2.45)	2.87 (2.12,3.67)	1.45 (1.13,1.89)
	East Asia	-1.54 (-1.66, -1.43)	16.39 (12.74, 20.80)	10.50 (7.85, 14.03)	-2.69 (-2.80,-2.58)	8.08 (6.30,10.26)	3.72 (2.80,4.96)	-4.01 (-4.16,-3.85)	315.73 (245.63,400.61)	99.15 (74.21,131.43)	-4.06 (-4.22,-3.90)	6.37 (4.96,8.08)	1.97 (1.48,2.61)
	High-income Asia Pacific	-2.91 (-3.02, -2.79)	51.47 (46.15, 56.81)	20.62 (17.39, 24.44)	-3.63 (-3.75,-3.51)	15.14 (13.59,16.61)	4.96 (4.20,5.88)	-4.90 (-5.04,-4.75)	370.29 (327.12,406.14)	84.16 (73.72,98.12)	-4.89 (-5.03,-4.75)	7.25 (6.45,7.93)	1.66 (1.45,1.93)
	High-income North America	0.65 (0.48, 0.81)	3.84 (3.66, 4.04)	4.45 (4.17, 4.75)	0.15 (0.03,0.26)	1.28 (1.23,1.34)	1.29 (1.22,1.38)	-0.33 (-0.42,-0.24)	33.44 (32.21,34.71)	29.17 (27.65,30.77)	-0.32 (-0.41,-0.23)	0.67 (0.64,0.69)	0.58 (0.55,0.61)
	North Africa and Middle East	-1.76 (-1.87, -1.66)	6.70 (5.22, 8.14)	3.74 (2.75, 4.74)	-1.86 (-1.99,-1.73)	3.59 (2.80,4.35)	1.94 (1.41,2.46)	-1.87 (-1.99,-1.75)	166.58 (130.17,202.40)	89.85 (65.61,113.93)	-1.88 (-2.01,-1.75)	3.33 (2.61,4.05)	1.79 (1.31,2.27)
	Oceania	-0.63 (-0.71, -0.55)	7.87 (4.62, 11.98)	6.57 (4.13, 10.21)	-0.72 (-0.79,-0.64)	4.05 (2.39,6.16)	3.27 (2.06,5.09)	-0.76 (-0.84,-0.68)	181.17 (106.51,277.40)	143.28 (90.62,222.91)	-0.80 (-0.88,-0.71)	3.53 (2.09,5.39)	2.76 (1.75,4.29)
	South Asia	-1.39 (-1.47, -1.31)	4.07 (3.29, 4.87)	2.63 (2.18, 3.14)	-1.52 (-1.61,-1.44)	2.14 (1.74,2.56)	1.33 (1.10,1.58)	-1.70 (-1.78,-1.63)	94.51 (76.41,113.06)	55.26 (45.70,66.03)	-1.69 (-1.77,-1.62)	1.88 (1.52,2.25)	1.10 (0.91,1.31)
	Southeast Asia	-1.90 (-2.05, -1.76)	5.41 (4.23, 6.50)	3.21 (2.62, 3.94)	-2.16 (-2.29,-2.03)	2.81 (2.21,3.37)	1.53 (1.26,1.88)	-2.53 (-2.67,-2.40)	122.56 (95.79,147.27)	59.51 (48.80,73.13)	-2.49 (-2.62,-2.37)	2.43 (1.91,2.92)	1.19 (0.98,1.46)
	Southern Latin America	-0.84 (-0.98, -0.70)	5.27 (4.55, 6.09)	3.78 (3.16, 4.47)	-1.19 (-1.33,-1.05)	2.51 (2.18,2.91)	1.63 (1.37,1.90)	-1.57 (-1.70,-1.45)	97.69 (84.96,112.54)	55.88 (47.33,64.78)	-1.56 (-1.69,-1.42)	1.96 (1.70,2.25)	1.13 (0.95,1.30)
	Southern Sub-Saharan Africa	-0.48 (-1.13, 0.17)	4.19 (3.54, 4.93)	2.86 (2.20, 3.73)	-0.31 (-0.95,0.33)	2.12 (1.79,2.50)	1.50 (1.16,1.95)	-0.37 (-1.05,0.31)	90.95 (76.65,107.18)	63.06 (48.18,82.49)	-0.28 (-0.92,0.36)	1.79 (1.51,2.11)	1.27 (0.97,1.65)
	Tropical Latin America	-0.86 (-0.92, -0.80)	5.45 (5.08, 5.84)	4.38 (4.02, 4.76)	-1.13 (-1.19,-1.06)	2.78 (2.58,2.97)	2.06 (1.90,2.23)	-1.35 (-1.41,-1.29)	115.87 (107.87,124.06)	79.93 (74.02,86.63)	-1.36 (-1.42,-1.30)	2.34 (2.17,2.50)	1.61 (1.49,1.74)
	Western Europe	-1.23 (-1.43, -1.03)	6.06 (5.62, 6.55)	3.84 (3.47, 4.26)	-2.15 (-2.27,-2.03)	2.46 (2.30,2.64)	1.22 (1.11,1.33)	-3.01 (-3.07,-2.95)	79.21 (74.71,84.18)	30.34 (28.28,32.56)	-2.97 (-3.04,-2.91)	1.60 (1.51,1.70)	0.62 (0.58,0.66)
	Western Sub-Saharan Africa	-1.16 (-1.25, -1.08)	3.22 (2.53, 3.89)	2.20 (1.64, 2.76)	-1.26 (-1.34,-1.19)	1.74 (1.37,2.09)	1.15 (0.86,1.45)	-1.31 (-1.38,-1.24)	75.93 (59.82,91.78)	49.50 (37.12,62.39)	-1.33 (-1.40,-1.27)	1.53 (1.21,1.85)	1.00 (0.75,1.25)

### Changes in the GBD of Gastric cancer in women aged 15–49 at socio-demographic index levels

A consistent gradient emerged when stratifying by SDI. High-SDI regions exhibited the largest reductions in DALYs (EAPC: -3.53) and deaths (EAPC: -3.52), indicating that more developed health systems, greater resource availability, and improved early detection likely contributed to these declines. High-income Asia Pacific, which also falls into a higher SDI category, showed particularly marked decreases in prevalence (from 51.47 to 20.62; EAPC: -2.91) and incidence (EAPC: -3.63), and had the steepest reductions in DALYs (EAPC: -4.90) and deaths (EAPC: -4.89) among all regions (**Table 1**).

In contrast, Low-SDI and Low-middle SDI regions saw more modest reductions in prevalence (EAPC: -1.50 and -1.25) and incidence (EAPC: -1.62 and -1.38), suggesting ongoing challenges such as limited access to advanced treatments, diagnostic services, and preventive interventions. However, these regions still demonstrated downward trends, indicating that even resource-constrained settings are experiencing some improvements in stomach cancer outcomes (**Table 1**).

### Regional burden of gastric cancer in women of reproductive age (15–49)

Among the 21 GBD regions, the highest initial prevalence in 1990 was observed in High-income Asia Pacific (51.47, 95% UI: 46.15–56.81), yet it experienced one of the steepest declines by 2021 (20.62, 17.39–24.44). Eastern Europe had an initial prevalence of 15.86 (15.12–16.64) and declined to 8.09 (7.11–9.09), reflecting an EAPC of -2.28. By contrast, High-income North America showed a slight increase in prevalence (EAPC: +0.65), rising from 3.84 (3.66–4.04) to 4.45 (4.17–4.75). Incidence remained essentially stable (EAPC: +0.15), while DALYs and deaths still declined modestly.

Regions such as Central Asia, Central Europe, and Eastern Europe showed large negative EAPCs in most metrics, indicating a considerable reduction in disease burden. For example, Central Asia’s prevalence EAPC was -2.88, with a drop in prevalence from 10.53 (9.74–11.39) to 4.22 (3.58–4.95), while its DALYs also fell sharply (EAPC: -3.17) (**Table 1**).

### Gastric cancer in women of reproductive age (15–49) in 204 countries and areas

In 2021, the global age-standardized prevalence (ASPR) of gastric cancer among females was 5.41 per 100,000 (95% UI: 4.78–6.18). East Asia had notably high prevalence, especially the Republic of Korea (28.73, 95% UI: 19.68–41.16) and Japan (17.83, 95% UI: 15.25–20.00). In contrast, prevalence remained below 1 per 100,000 in some Middle Eastern (e.g., Kuwait, 0.59, 95% UI: 0.44–0.77) and sub-Saharan African settings (e.g., Nigeria, 0.86, 95% UI: 0.51–1.36).

**Figure 1:**
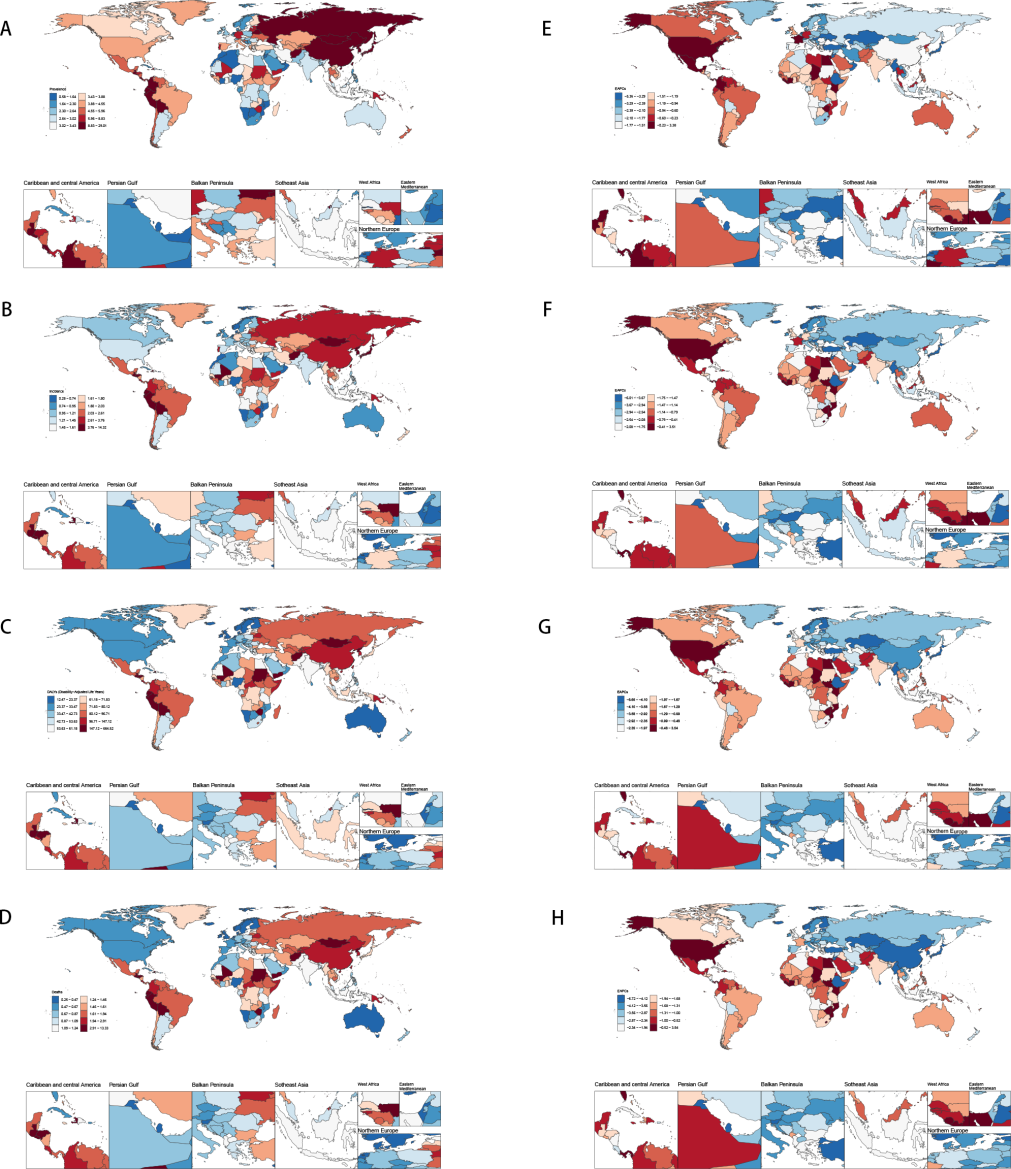
Disease burden of stomach cancer in women of reproductive age in 204 countries and regions worldwide in 2021 **(A)** Age-standardized incidence; **(B)** Age-standardized prevalence; **(C)** Age-standardized DALYs; **(D)** Age-standardized death; **(E)** EAPC of age-standardized incidence; **(F)** EAPC of age-standardized prevalence; **(G)** EAPC of age-standardized DALYs; **(H)** EAPC of age-standardized death.

Between 1990 and 2021, the Estimated Annual Percentage Change (EAPC) generally indicated declining trends. Maldives (EAPC: -5.31, 95%UI: -5.53-5.08) and Kuwait (EAPC: -4.28, 95%UI: -5.12,-3.44) showed some of the steepest declines. Many countries had moderate negative EAPCs, while a few reported near-zero or slightly positive EAPCs, suggesting slower or minimal declines in prevalence. Overall, these findings indicate a global downward trend in prevalence, though notable heterogeneity persists across countries (**Figures 1A, E**).

Global incidence of stomach cancer among females decreased from 4.61 per 100,000 (95% UI: 4.11–5.15) in 1990 to 2.13 (95% UI :1.89–2.41) in 2021. Despite this overall decline, considerable heterogeneity exists across countries. For example, China’s incidence fell from 8.20 (95% UI: 6.36–10.46) to 3.73 (95% UI: 2.78–5.02), while other nations—such as the Democratic People’s Republic of Korea and Taiwan—also experienced reductions, though the magnitude of change varied. The global EAPC was –2.56, indicating a consistent downward trend; however, country-specific EAPCs spanned a wide range. Some countries exhibited increasing trends (e.g., Lesotho with an EAPC of 3.48 [95% CI: 2.59–4.37] and Zimbabwe with 3.27 [2.05–4.51]), whereas others, such as Maldives, showed sharp declines (EAPC –5.95 [–6.22 to –5.68]). These divergent trends underscore the need for region-specific interventions to address the unique epidemiological patterns observed in different settings (**Figures 1B, F**).

DALYs dropped from 173.76 (153.39–195.06) to 71.45 (63.78–79.53) per 100,000 females, showing a global EAPC of -3.00, reflecting a substantial reduction in disease burden over time. East Asian countries (e.g., China, Republic of Korea, Japan), again experienced large declines in DALYs—some exceeding 70%—and many high-income settings (e.g., Western Europe, North America) showed moderate to steep negative trends, certain regions (e.g., parts of sub-Saharan Africa and select Pacific island nations) demonstrated slower progress or even rising DALYs (e.g., Zimbabwe, Lesotho) (**Figures 1C, G**).

Overall, there was a global decline in stomach cancer incidence, prevalence, and DALYs among females from 1990 to 2021; however, substantial geographic disparities in both absolute rates and EAPCs persist. While many countries—especially those with higher SDI—are experiencing steep declines, others present slower reductions or even increasing trends. These findings underscore the importance of region-specific strategies, including targeted screening, better management of risk factors, and continued research into local determinants of stomach cancer to further reduce the global disease burden. Thus, although the global burden is declining, disparities in magnitude and pace of change remain a central challenge (**Figure 1**) (Details on the global burden data of gastric cancer in women of reproductive age for 204 countries and regions can be found in **Appendix Table 1** in **Supplementary Material**).

### Stomach cancer GBD trends in women aged 15–49 over time

The global prevalence and incidence rates dropped gradually, particularly in high-SDI and high-middle SDI regions. High-SDI regions initially exhibit higher prevalence and incidence rates, especially among middle-aged and older populations, although they also showed a more rapid decline over time. In contrast, low-SDI and low-middle SDI regions generally had lower baseline levels, though in some age groups the rates remained relatively stable or showed only minimal fluctuations. Among all age groups, the younger populations (15–19 years) exhibit low prevalence and incidence, while older populations represent a higher risk (**Appendix Figures A1**-**A8**).

**Figure 2:**
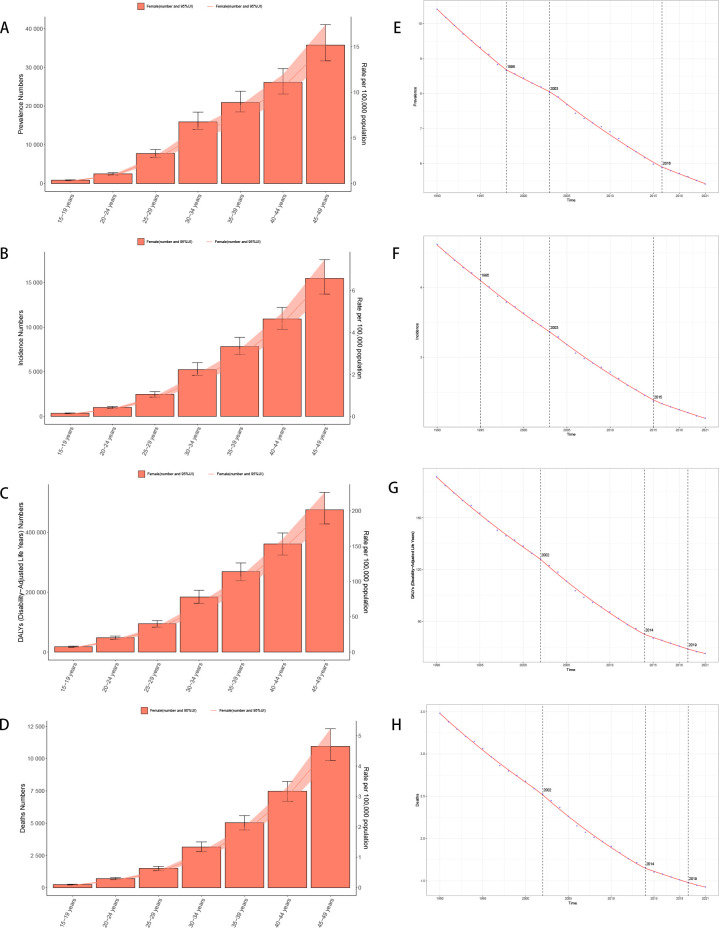
Trend of disease burden of gastric cancer in women of reproductive age and Joinpoint regression **(A)** Prevalence; **(B)** Incidence; **(C)** DALYs; **(D)** Death; **(E)** Joinpoint regression of prevalence; **(F)** Joinpoint regression of incidence; **(G)** Joinpoint regression of DALYs; **(H)** Joinpoint regression of death.

The burden of female stomach cancer increases progressively with age. In the 15–19-year age group, incidence, deaths, and DALYs (Disability-Adjusted Life Years) remain relatively low, but they rise substantially from the age of 30 onward, peaking in the 45–49-year group. For instance, the incidence rate in women aged 15–19 is around 0.11 per 100,000, whereas among those aged 45–49, it reaches approximately 6.57 per 100,000—underscoring the greater disease burden at older ages (**Figures 2A-D**).

These findings highlight the role of healthcare system capacity in shaping epidemiological patterns and underscore the widening disparities between regions. Such heterogeneity across SDI levels provides important context for tailoring prevention and screening strategies.

The Joinpoint Analysis of stomach cancer trends in women aged 15-49 (Figures E to H) shows a general decline in incidence, mortality, and DALYs across all age groups, with more significant reductions in older cohorts (40–49 years) (**Figures 2E-H**).

### Age-period-cohort analyze of gastric cancer in women aged 15–49

Age Effects: As women age, the burden of gastric cancer increases significantly, as seen in all four metrics: prevalence, incidence, deaths, and DALYs. In particular, the rates for women in the 40–49 age group are consistently higher than that for younger age groups. In the DALY metric, rates significantly increase from younger groups (such as 15–19 and 20–24) to older groups (40–44 and 45–49), which reinforces the well-established understanding that older age is a major risk factor for gastric cancer.

Period Effects: Over time, from the 1990s to the present day, there is a noticeable decline in the rates of gastric cancer in many regions, particularly among younger populations. In the period effect analysis, we can observe that the rate of deaths, incidence, and DALYs has reduced over the years, with minor fluctuations.

**Figure 3:**
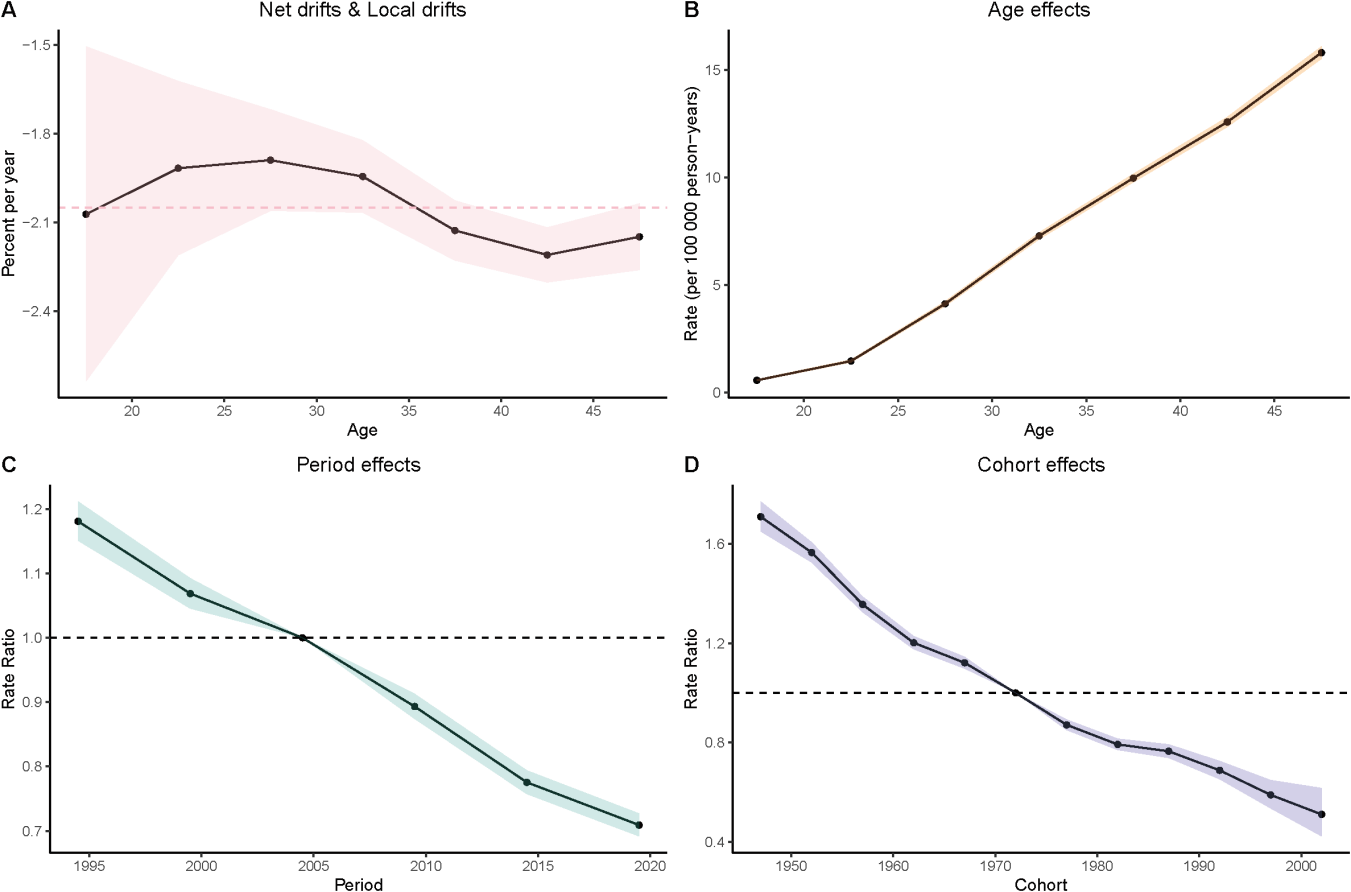
Age–period–cohort (APC) analysis of prevalence. **(A)** Local drifts and net drifts; **(B)** Age effects; **(C)** Period effects; **(D)** Cohort effects.

**Figure 4:**
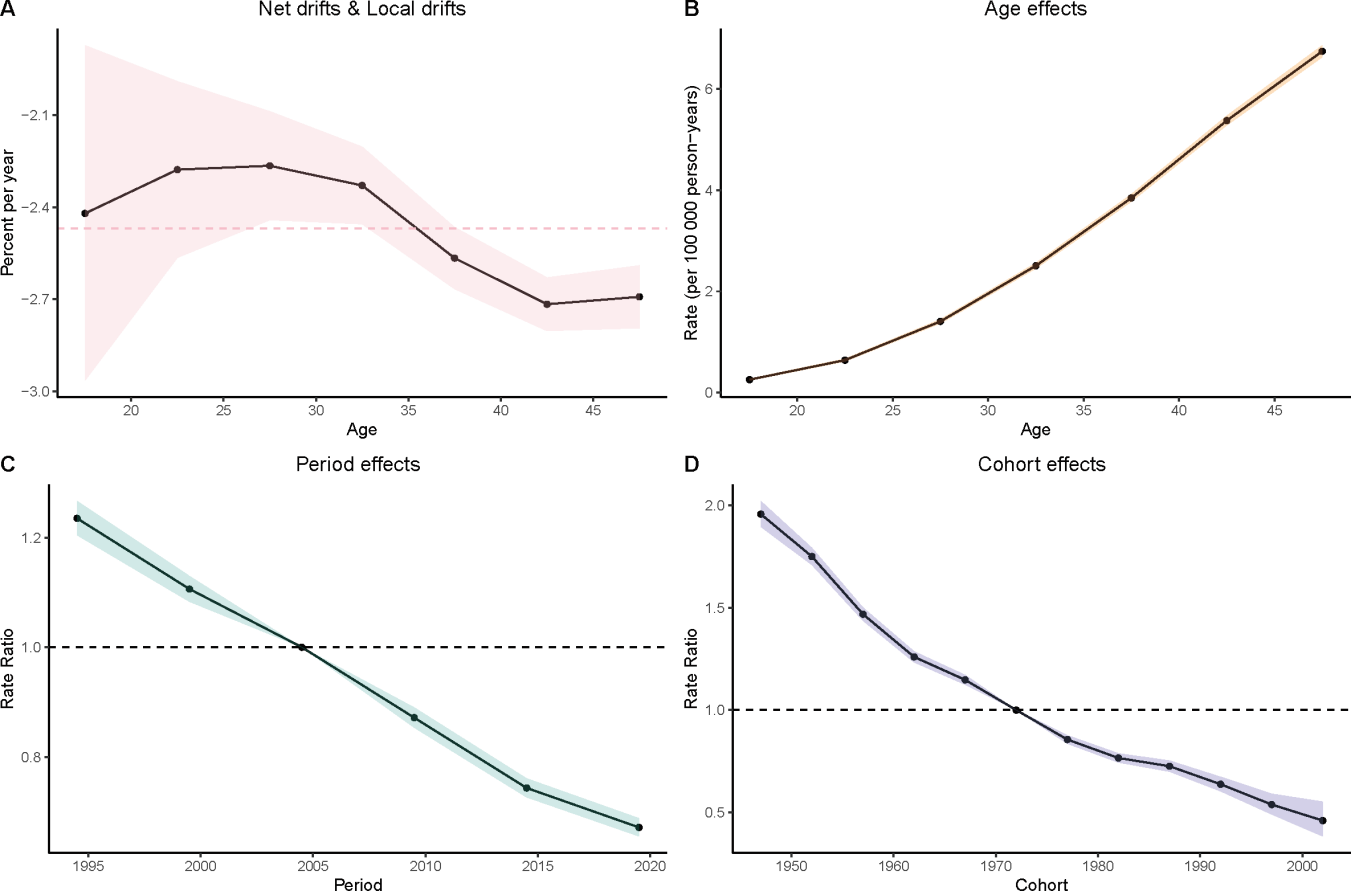
Age–period–cohort (APC) analysis of incidence. **(A)** Local drifts and net drifts; **(B)** Age effects; **(C)** Period effects; **(D)** Cohort effects.

**Figure 5:**
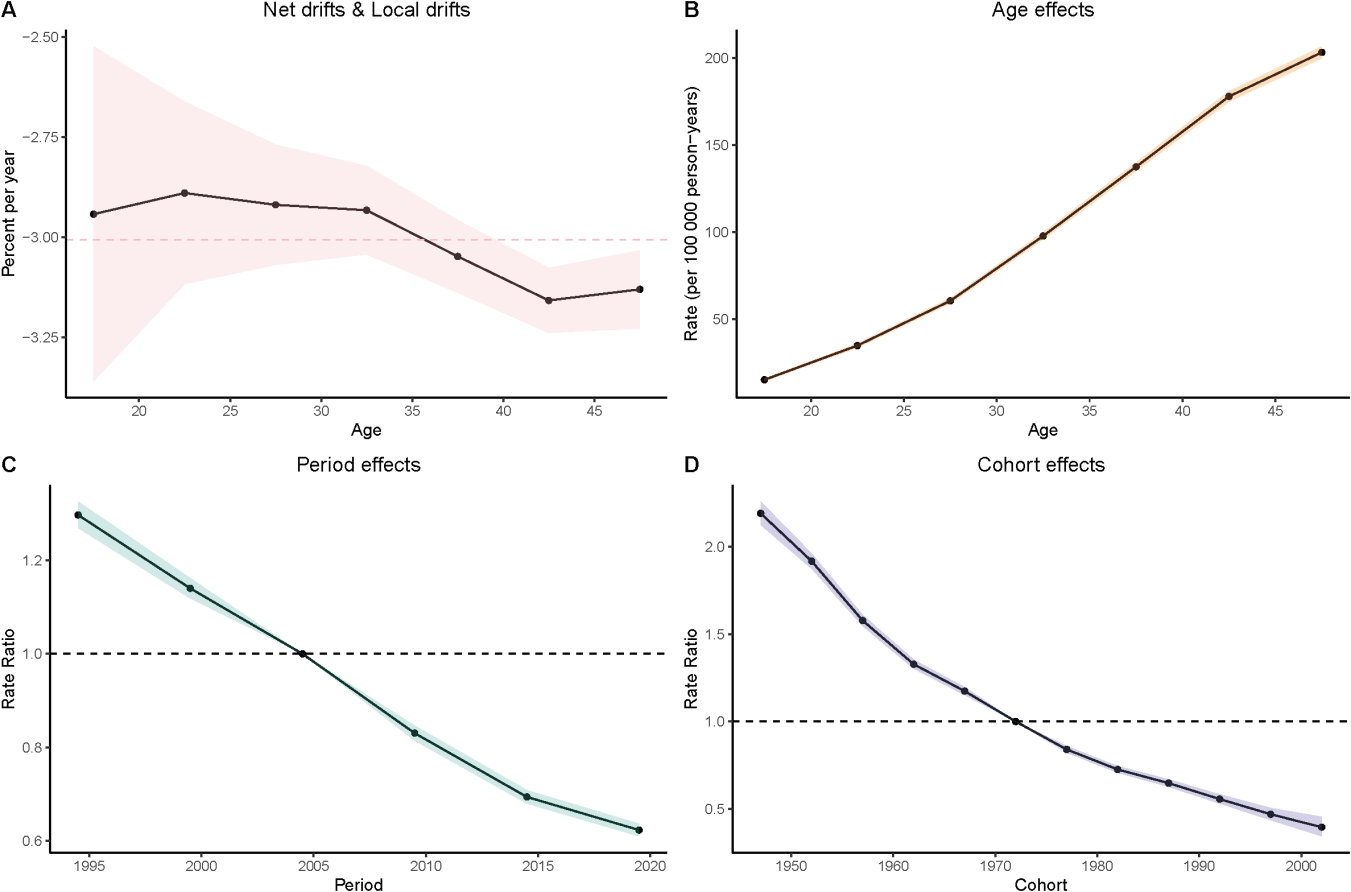
Age–period–cohort (APC) analysis of DALYs. **(A)** Local drifts and net drifts; **(B)** Age effects; **(C)** Period effects; **(D)** Cohort effects.

**Figure 6:**
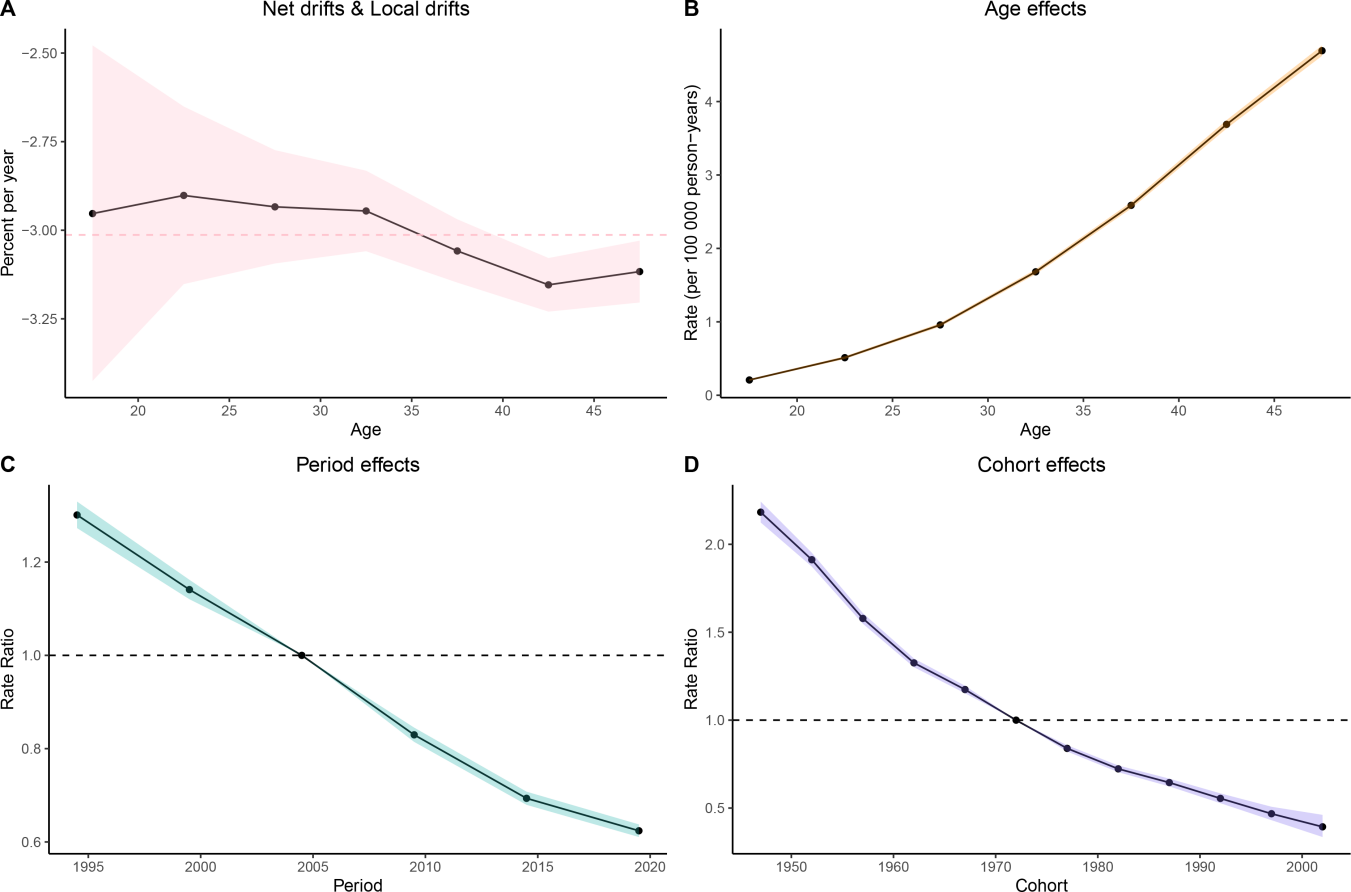
Age–period–cohort (APC) analysis of deaths. **(A)** Local drifts and net drifts; **(B)** Age effects; **(C)** Period effects; **(D)** Cohort effects.

Cohort Effects: The cohort analysis reveals how specific generations are impacted by their historical exposures to risk factors. Older cohorts (such as those born before the 1970s) show higher rates of gastric cancer in comparison to more recent generations, especially in the incidence and prevalence measures. This suggests that earlier cohorts may have had more exposure to dietary or environmental risk factors, such as higher salt consumption or Helicobacter pylori infections, which have been linked to higher gastric cancer risks. The more recent cohorts show a modest decline in these measures, possibly indicating that improved health awareness and changes in dietary habits are beginning to yield positive effects (**Figures 3**–**6**).

### SDI-related analysis

The relationship between SDI and the age-standardized incidence, prevalence, DALYs and death varies considerably across regions. With increasing SDI, prevalence and incidence tend to rise initially. DALYs and deaths also increase, peaking at an SDI of approximately 0.5, after which the burden declines as SDI continues to rise further.

**Figure 7:**
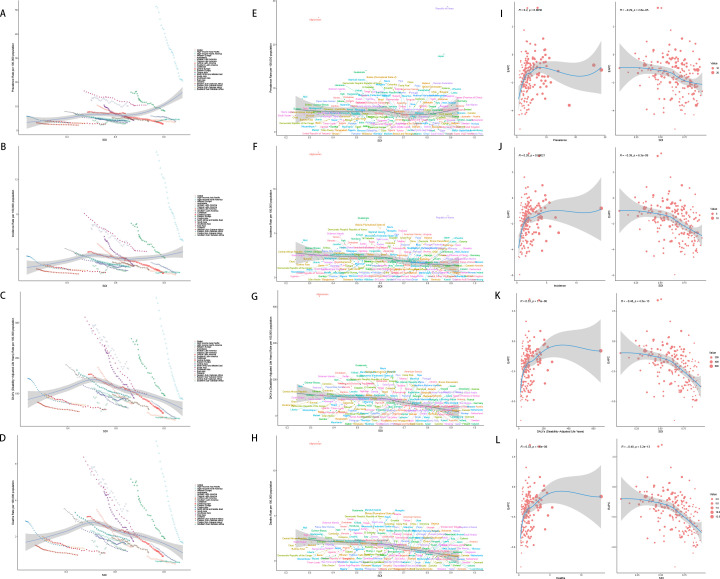
SDI analysis **(A)** Incidence rates in 21 regions; **(B)** Prevalence rates in 21 regions; **(C)** DALYs in 21 regions; **(D)** Deaths in 21 regions; **(E)** Incidence rates in 204 countries; **(F)** Prevalence rates in 204 countries; **(G)** DALYs in 204 countries; **(H)** Deaths in 204 countries; **(I)** EAPC of incidence rates; **(J)** EAPC of prevalence rates; **(K)** EAPC of DALYs; **(L)** EAPC of deaths.

For prevalence, in 204 countries, the correlation between the prevalence and SDI is weak and not statistically significant (r =-0.1089, p 1.211e-01). In 21 GBD regions, however, there is a significant positive correlation between them (r=0.2391, p=1.490e-10). For incidence, in 21 GBD regions, the correlation between the incidence and SDI is weak and not statistically significant (r=-0.0520, p=1.681e-01), however, in 204 countries, there is a moderate negative correlation between the incidence and SDI, which is statistically significant (r=-0.3245, p=2.498e-06). There are negative correlations between SDI and mortality in the 21 GBD regions and 204 countries, meaning that as SDI increases, the gastric cancer mortality decreases. This relationship is highly statistically significant, as the p-value is much smaller than 0.05. The same goes with the DALYs rate (**Figures 7A-H**).

However, the EAPC of prevalence, incidence, DALYs and death show a downward trend, which means as the SDI increases, the disease burden is getting better (**Figures 7I-K**).

### Decomposition analysis

Decomposition analysis revealed that population growth, aging, and epidemiological changes all shaped the gastric cancer burden among women of reproductive age. Globally, epidemiological improvements reduced the burden, whereas aging increased it. Population growth had an overall negative impact but showed positive effects in some regions.

Across SDI strata, epidemiological shifts consistently drove declines, while aging exerted the opposite effect. In High- and High–middle-SDI regions, population growth and aging partially offset these gains, whereas in lower-SDI settings, the net benefit of epidemiological changes remained more evident.

**Figure 8:**
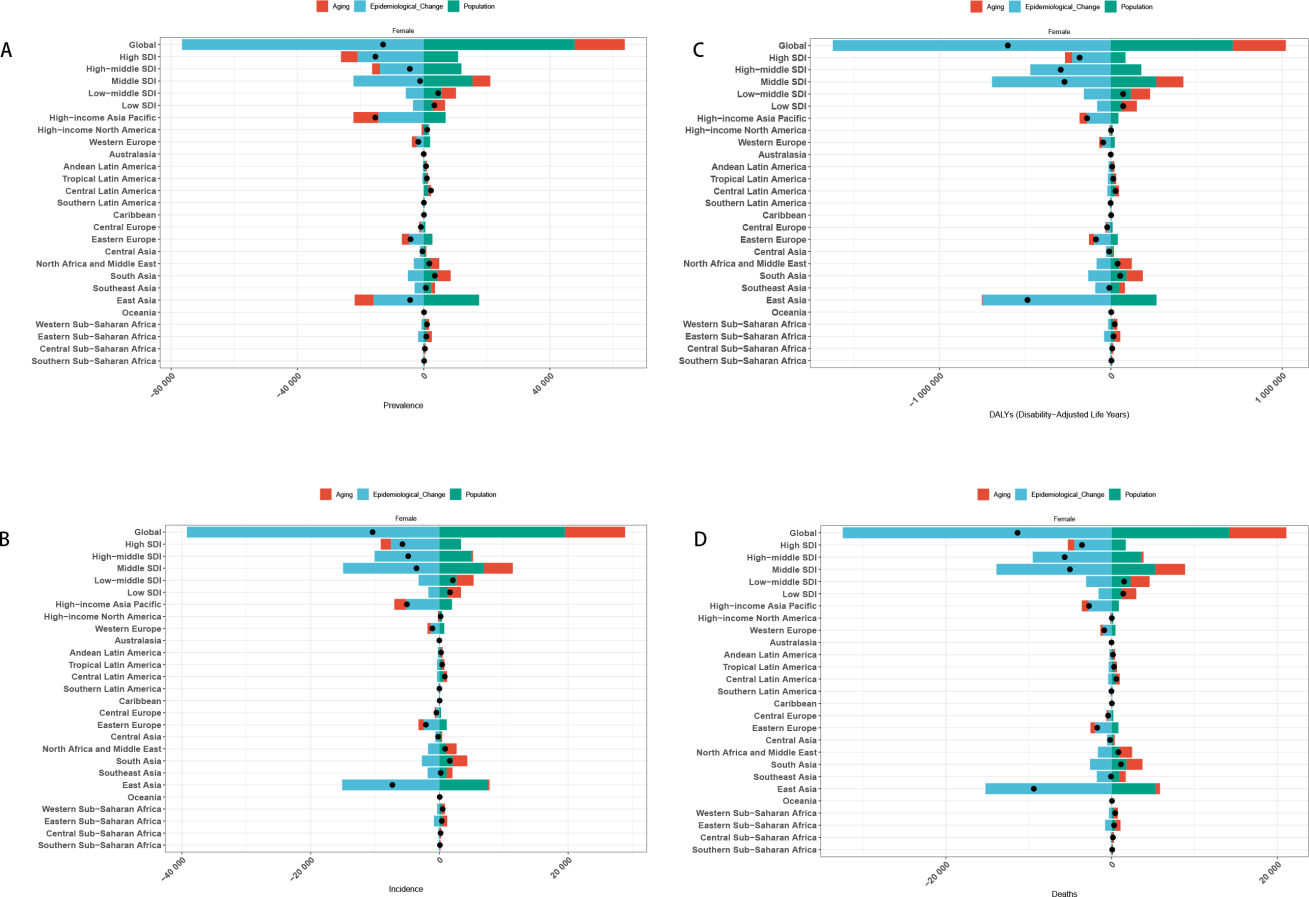
Decomposition analysis results [**(A)** Incidence in Global+ 5 SDI regions+21 GBD regions; **(B)** Prevalence in Global+ 5 SDI regions+21 GBD regions; **(C)** DALYs in Global+ 5 SDI regions+21 GBD regions; **(D)** Death in Global + 5 SDI regions+21 GBD regions].

At the regional level, heterogeneity persisted. High-income Asia Pacific, Western Europe, North America, Eastern Europe, and East Asia experienced a positive contribution from population growth, counterbalancing epidemiological gains. In other regions, declining trends were largely attributable to epidemiological improvements (**Figure 8**).

### Predictive analysis

**Figure 9:**
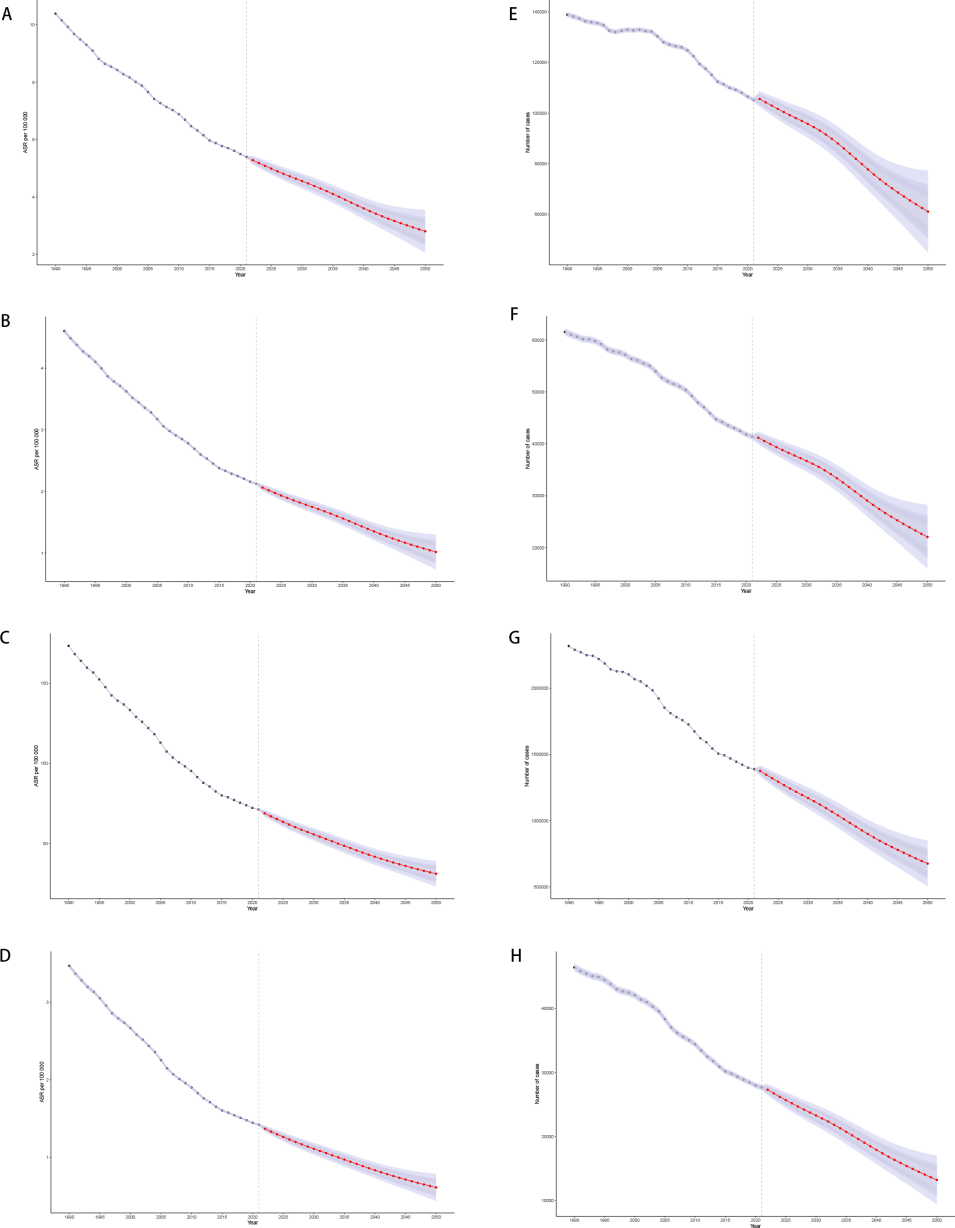
Predictive analysis results of 2050 [**(A)** Age-standardized incidence; **(B)** Age-standardized prevalence; **(C)** Age-standardized DALYs; **(D)** Age-standardized Death; **(E)** Actual incidence; **(F)** Actual prevalence; **(G)** Actual DALYs; **(H)** Actual Death].

Predictive analysis suggests that from 2022 to 2050, the global burden of gastric cancer among females of reproductive age will continue to decline. By 2050, the age-standardized incidence, prevalence, DALYs, and mortality are projected to fall to 2.81 (95% UI: 2.06–3.56), 1.02 (0.73–1.30), 31.15 (23.13–39.17), and 0.61 (0.43–0.78) per 100,000 persons, respectively. This corresponds to an estimated 22,076 new cases, 61,045 prevalent cases, 676,399 DALYs, and 13,228 deaths globally (**Figure 9**).

Age-specific projections reveal consistent downward trends across all age groups, although the reductions are less pronounced in older women, reflecting the persistent impact of demographic aging (**Appendix Figures A9–A14** in **Supplementary Material**). Overall, these forecasts highlight both the progress made in controlling gastric cancer and the need for sustained, region-specific strategies to further reduce the burden among women of reproductive age.

### Cross-country inequality analysis

**Figure 10:**
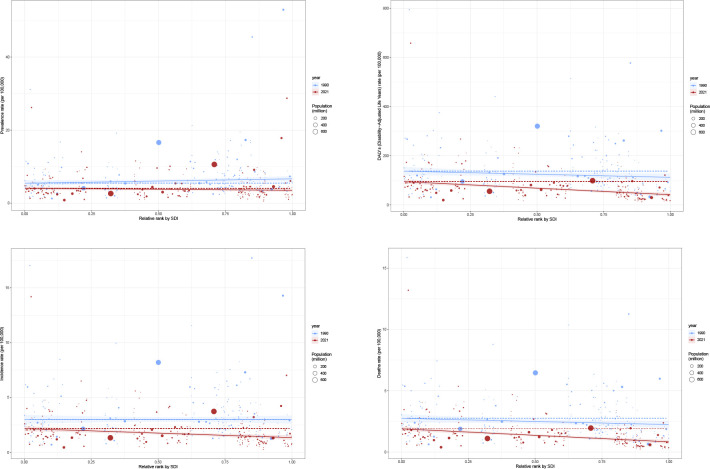
Health inequality regression curves for 1990 and 2021 [**(A)** Prevalence; **(B)** Incidence; **(C)** DALYs; **(D)** Death].

SII of prevalence, incidence for 1990 is 1.19 (95% CI: -0.17, 2.55), -0.02(95% CI: -0.70, 0.66), suggesting that in 1990, regions with lower SDI experienced a higher disease burden, whereas higher SDI regions had a lower burden, indicating greater health inequality, but there remains uncertainty in this inequality trend and a possible lack of statistical significance. SII of prevalence, incidence for 2021 is -0.54 (95% CI: -1.46, 0.38), -0.82(95% CI: -1.22, -0.42) indicates a potential reversal in health inequality, where higher SDI regions may now have a higher disease burden, also, shift in inequality may not be statistically significant. However, the SII of DALYs for 1900(-25.07(95% CI: -54.15, 4.02)) and 2021(-53.17(CI: -67.79, -38.55)) suggests that from 1990 to 2021, health inequality in DALYs has increased, the 1990 estimate had uncertainty, but by 2021, the data strongly indicates that low SDI regions bear a disproportionately high burden of DALYs. This suggests that despite overall global health improvements, lower SDI regions have not benefited equally, and disparities have deepened (**Figure 10**).

### Population attributable fraction of death

The decomposition analysis of attributable risk factors for gastric cancer among females of reproductive age revealed significant contributions from behavioral and dietary risks, particularly smoking, high sodium intake, and other dietary factors. The proportion of deaths attributed to these risk factors varied across different SDI regions, with lower SDI regions exhibiting a higher burden from dietary and behavioral risks​.

**Figure 11:**
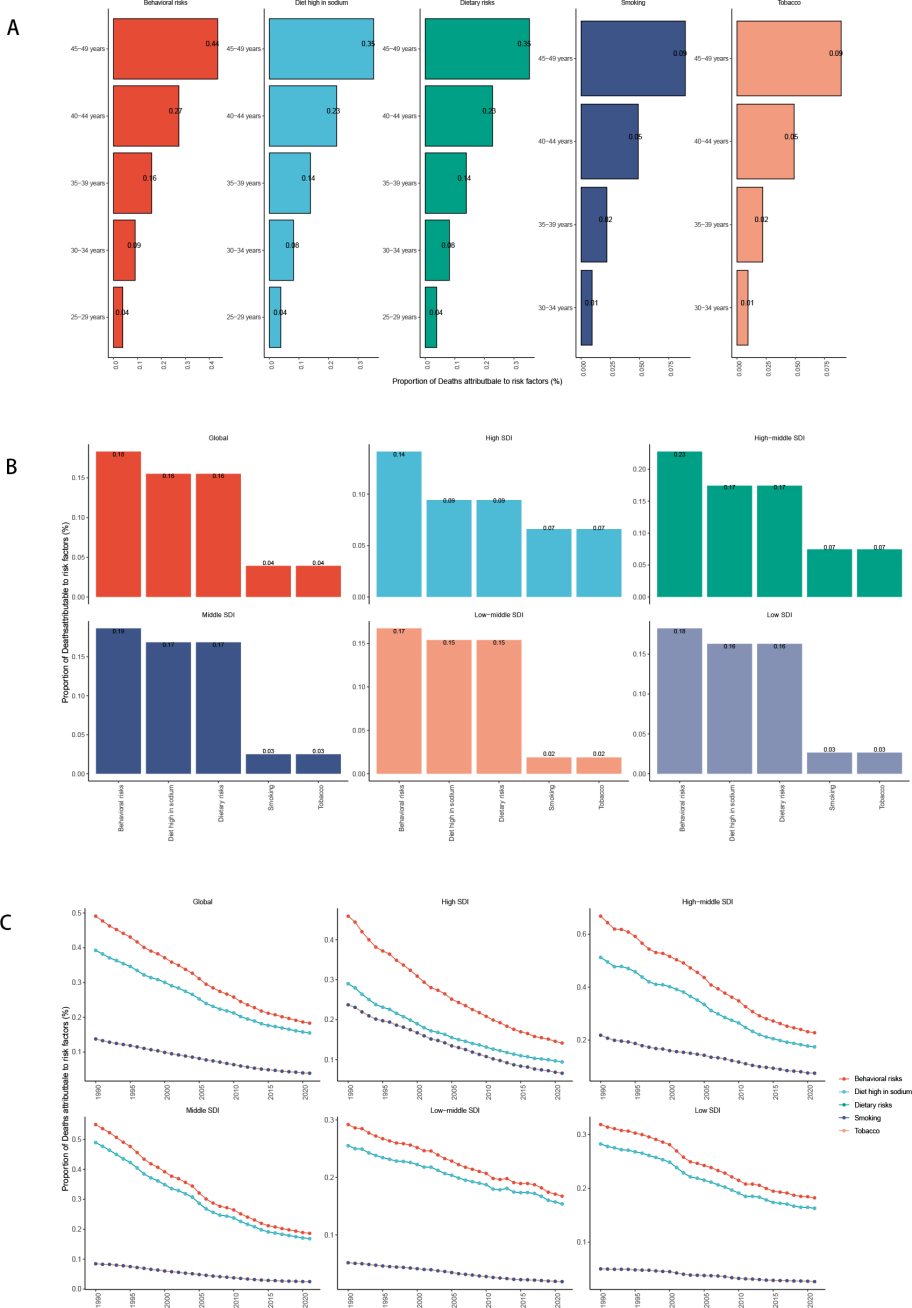
Risk factors for death in the global and five SDI regions [**(A)** Risk factors for different age groups; **(B)** Risk factors for in 2021; **(C)** Temporal trends of risk factors].

Temporal trends indicate that while the burden of behavioral risks has gradually declined over the past decades in high and high-middle SDI regions, it remains persistent or has even increased in low and low-middle SDI regions. The global reduction in smoking-related risks has been counterbalanced by the sustained impact of high sodium consumption and other dietary risks​. Over time, the disease burden attributable to different risk factors has shown an overall decreasing trend, with behavioral risks exhibiting the most significant decline (**Figure 11**, **Appendix Figures A15, A16** in **Supplementary Material**).

### Frontier analysis

To investigate the gastric cancer (GC) burden among females of reproductive age, a frontier analysis of age-standardized death rates (ASDR) was conducted across 204 countries and territories from 1990 to 2019. This analysis, which uses ASDR as a key metric for understanding the impact of GC, delineates countries and regions along a frontier line based on their respective SDI levels. The top five countries with the greatest effective difference from the frontier, included Afghanistan (657.9), Yemen (170.7), Central African Republic (102.8), and Chad (89.1). Conversely, the top five countries with the lowest ASDR within their development spectrum included Somalia, Switzerland (17.3), Sweden (18.9), Norway (16.7), and Luxembourg (15.8). Developed countries (e.g., USA, Canada, Germany) have higher SDI values (above 0.8) and lower DALYs.

**Figure 12:**
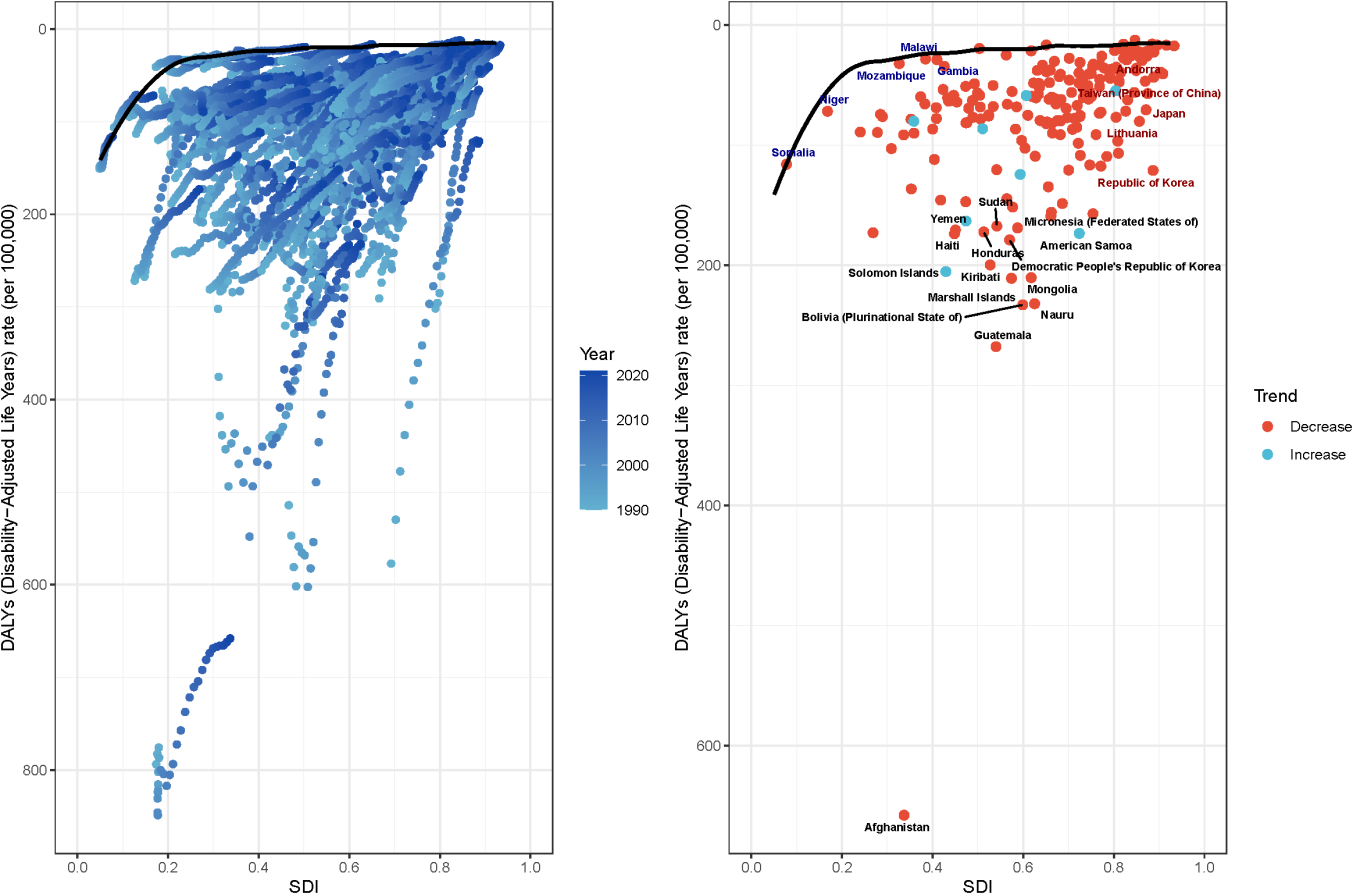
Frontier analysis of based on SDI and ASDR from 1990 to 2021. **(A)** Each dot represents a country where that line performs best (i.e., the case with the lowest disease burden, i.e., the frontier frontiers). The colour of the dots from light blue to dark blue represents how the disease burden changes as the year progresses from 1990-2021. A country’s effective distance from frontiers is defined as the gap between its observed and potentially realisable disease burden; this gap can be reduced or eliminated by using the country’s socio-demographic resources. **(B)** Dots (with black autosomes) represent the 15 countries and territories with the largest differences from the frontiers. Dots (with blue autosomes) represent the 5 countries with the smallest differences from the frontier among low SDI countries (<0.50). Dots (with red autosomes) represent the 5 countries with the largest differences from the frontier among high SDI countries (>0.85). A red dot represents a country with a decreasing burden of disease, and a blue dot represents a country with an increasing burden of disease.

Developing or low-income countries (e.g., Sub-Saharan Africa, South Asia) had lower SDI values (below 0.5) and higher DALYs. This represents the gap between actual DALYs and the best possible health outcomes for a given SDI. Countries such as Maldives, Singapore, and Australia performed close to their expected best health outcomes. By contrast, countries such as Afghanistan, Sudan, and Guinea-Bissau exhibited much worse health outcomes than expected for their SDI level (**Figure 12**; Specific data are presented in **Appendix Table 2** in **Supplementary Material**).

The frontier analysis revealed extreme outliers, such as Afghanistan, where the observed burden of gastric cancer far exceeds the level expected given its national SDI. This stark disparity likely stems from factors not fully captured by the composite SDI metric. Afghanistan has endured decades of armed conflict, which has systematically destroyed healthcare infrastructure, displaced populations, and severely limited access to basic sanitation, nutritious food, and cancer care. Chronic food insecurity and high prevalence of H. pylori infection, fueled by poor living conditions, likely compound the risk. This situation highlights that in settings of profound fragility and humanitarian crisis, the standard socioeconomic and healthcare drivers of disease burden are overshadowed by the overwhelming effects of conflict and instability, leading to catastrophic health outcomes that conventional models struggle to predict.

## Discussion

### Key findings

This study presents a comprehensive assessment of the global, regional, and national burden of gastric cancer among women of reproductive age (15–49 years) from 1990 to 2021, based on data from the Global Burden of Disease (GBD) 2021 study. The findings demonstrate a significant overall decline in age-standardized prevalence, incidence, disability-adjusted life years (DALYs), and mortality rates from 1990 to 2021, particularly in high-SDI regions. However, notable disparities persist, particularly in low- and middle-SDI countries, where reductions have been more modest. These findings highlight the interplay between socioeconomic development, healthcare access, and epidemiological transitions in shaping global gastric cancer trends.

### Comparisons with prior studies

Female patients with advanced gastric cancer were significantly younger and often exhibit distinct histological characteristics compared to their male counterparts, especially in those under 45 years (13). Notably, young female patients with AGC, particularly those with signet ring cell carcinoma (SRC), show poorer overall survival rate. The expression of estrogen receptor-beta (ER-β) differed between females and males with SRC, suggesting a potential role for hormonal factors in prognosis. In a study conducted in Finland, the onset of intestinal-type gastric cancer occurred earlier in men than in women, whereas diffuse gastric cancer showed the opposite pattern, with a higher prevalence in younger female populations compared with older age groups.

### Potential explanations and mechanisms

Pregnancy and delivery processes in young females may influence the growth of gastric cancer (GC), making regular screening particularly crucial for women of reproductive age. Physiological changes during pregnancy, including immunosuppression, hormonal fluctuations, and altered gastric motility, may create a microenvironment conducive to tumor progression. For instance, PSG1 (Pregnancy specific beta-1-glycoprotein 1), an immunoglobulin highly expressed in several kinds of cancers, involves in the regulation of placental growth factor (PGF), TGF-β-mediated vascular endothelial growth factor (VEGF) and the activation and proliferation of T-cell (14). Studies suggest that estrogen may have a protective effect against gastric carcinogenesis (13, 15); however, hormonal shifts during pregnancy, such as increased progesterone levels, could modulate immune responses and promote tumor cell proliferation. Currently, research specifically addressing the impact of elevated progesterone levels during pregnancy on immune modulation and tumor cell proliferation, as well as the alterations in Helicobacter pylori infection patterns and gastric acid secretion leading to gastric mucosal damage and increased gastric cancer susceptibility, remains limited. However, case reports and clinical analyses have suggested that pregnancy-associated gastric cancer presents unique diagnostic and therapeutic challenges, which may be linked to these factors (16). Additionally, pregnancy induces immune tolerance to support fetal development, which can weaken the body’s ability to fight H. pylori infection, potentially exacerbating gastric mucosal damage. Pregnancy-induced physiological changes—such as hormonal fluctuations, altered immune responses, and changes in gastric acid secretion—can create an environment that favors *H. pylori* colonization, weakens gastric mucosal defense, and increases the risk of gastric mucosal damage and potentially gastric cancer (17).

While this research quantify population-level trends and do not capture tumor subtype or metastatic sites, complementary mechanistic data help interpret several age-specific patterns observed here (e.g., poorer outcomes in younger women and the prominence of diffuse/SRC histology). A single-cell study of ovarian metastases from gastric cancer reported that estrogen-responsive (ER-positive) ovarian fibroblasts secrete midkine (MDK) under estrogen stimulation, which engages LRP1 on gastric cancer cells and promotes migration, invasion, and metastatic colonization (18). Taken together, these findings suggest that, in premenopausal women, estrogen-driven tumor–stroma interactions could plausibly contribute to aggressive biology and ovarian involvement, offering a mechanistic hypothesis that aligns with our epidemiologic observations while warranting validation in integrated clinical–molecular datasets.

Moreover, the increased metabolic demand and potential micronutrient deficiencies, particularly iron and vitamin B12, associated with pregnancy could further compromise gastric mucosal integrity, leading to chronic inflammation and potential malignant transformation. A study in Bangladesh found that 29.8% of pregnant women were vitamin B12 deficient, while iron deficiency was also prevalent, leading to inflammation-related conditions (19).

### Policy implications

Given these risks, routine gastric cancer screening, particularly in women with a family history of GC, persistent gastrointestinal symptoms, or known Helicobacter pylori infection, is critical. Early detection through non-invasive methods such as serum pepsinogen testing, fecal occult blood tests, and gastroscopy could significantly improve prognosis in this population.

### Lifecourse hormonal evidence

Furthermore, among non-pregnant women, the global burden of gastric cancer continues to rise in conjunction with advancing age. Study suggests that hormonal changes over a woman’s lifetime may play a protective role against gastric cancer, and the risk increases with aging and hormonal decline (20). A large European cohort study (EPIC) (21) found that women who had late first pregnancies (>26 years old) had a decreased risk of gastric non-cardia cancer, but those who underwent bilateral ovariectomy had an increased risk, indicating that hormonal changes with aging and menopause may influence gastric cancer risk differently by tumor location. A case-control study in Japan found that higher cumulative estrogen exposure (longer reproductive years) was associated with a lower risk of gastric cancer.

Additional population-based evidence further supports the role of reproductive and hormonal factors in modulating gastric cancer risk. In the Singapore Chinese Health Study, a prospective cohort of over 34,000 women, later age at menopause, longer duration of menstrual cycling, and use of exogenous hormones such as oral contraceptives and hormone replacement therapy were all associated with significantly reduced gastric cancer risk (22). These findings lend epidemiologic support to the hypothesis that cumulative estrogen exposure and exogenous hormone use exert protective effects against gastric carcinogenesis, complementing the mechanistic and age-specific patterns discussed above.

However, women with early menopause and fewer pregnancies appeared to have a higher risk, particularly for undifferentiated gastric cancer, supporting the possible role of hormonal aging in cancer susceptibility (23).

### Declining global burden with socioeconomic disparities

The study observed a global decline in gastric cancer burden, with age-standardized prevalence decreasing from 10.42 per 100,000 in 1990 to 5.41 per 100,000 in 2021 (EAPC: -2.11), and incidence falling from 4.61 per 100,000 to 2.13 per 100,000 (EAPC: -2.56). Similarly, DALYs and mortality exhibited substantial reductions (EAPCs of -3.00 and -3.02, respectively), reflecting advancements in early detection, improved treatment modalities, and preventive measures, particularly in high-income regions. These findings align with previous research indicating a sustained global downward trend in gastric cancer incidence and mortality, largely driven by decreasing Helicobacter pylori (H. pylori) prevalence, improved hygiene, and dietary modifications.

When stratified by Socio-Demographic Index (SDI) levels, high-SDI regions exhibited the most substantial reductions in gastric cancer burden. For instance, High-income Asia Pacific saw the steepest declines, with prevalence dropping from 51.47 per 100,000 to 20.62 per 100,000 (EAPC: -2.91), and DALYs decreasing at an annual rate of -4.90%. The high-SDI regions’ success can be attributed to advanced healthcare infrastructure, widespread endoscopic screening programs, and lifestyle modifications. Conversely, low-SDI and lower-middle-SDI regions experienced slower declines, with prevalence EAPCs of -1.50 and -1.25, respectively. These modest reductions suggest that limited healthcare resources, diagnostic delays, and persistent risk factors such as H. pylori infection and high-sodium diets continue to hinder progress in these regions.

### Regional burden: persistent disease burden in specific areas

Among the 21 GBD regions, East Asia, Eastern Europe, and Central Asia historically exhibited the highest prevalence and incidence rates of gastric cancer in women aged 15–49. While some regions, such as Central Asia (EAPC: -2.88) and Eastern Europe (EAPC: -2.28), have shown notable declines, the overall disease burden remains disproportionately high compared to global averages. In contrast, High-income North America experienced a slight increase in prevalence (EAPC: +0.65), despite stable incidence rates, potentially due to improved detection, population aging, or changes in dietary patterns.

At the national level, South Korea (28.73 per 100,000) and Japan (17.83 per 100,000) had the highest prevalence rates in 2021, despite notable reductions in incidence and mortality. Countries such as Maldives (EAPC: -5.31) and Kuwait (EAPC: -4.28) exhibited the steepest declines, reflecting the effectiveness of health interventions, dietary modifications, and targeted cancer control policies. Conversely, certain Sub-Saharan African nations (e.g., Lesotho, Zimbabwe) experienced increasing trends (EAPC: +3.48 and +3.27, respectively), emphasizing the need for region-specific cancer prevention and treatment strategies.

### Age-specific trends: gastric cancer burden increases with age

The study confirms that gastric cancer burden increases progressively with age, with incidence, mortality, and DALYs peaking in the 45–49 age group (6.57 per 100,000). The burden remains relatively low in younger women (e.g., 15–19 years, 0.11 per 100,000), reinforcing the understanding that cumulative exposure to risk factors, biological aging, and immune system changes contribute to disease progression over time. Joinpoint regression analysis further reveals more significant reductions in gastric cancer burden among older reproductive-age cohorts (40–49 years), likely due to increased access to early detection and treatment options.

### Future projections: continued decline but persisting disparities

The predictive analysis estimates that by 2050, global age-standardized incidence, prevalence, DALYs, and mortality will further decrease, with projected incidence at 2.81 per 100,000, prevalence at 1.02 per 100,000, DALYs at 31.15 per 100,000, and mortality at 0.61 per 100,000. While these reductions reflect continued advancements in healthcare accessibility, treatment efficacy, and risk reduction strategies, disparities are expected to persist, particularly in low-SDI countries, where gastric cancer burden will remain disproportionately high.

### Health inequality and socioeconomic disparities

Despite global improvements, cross-country health inequality remains a challenge. The Standardized Inequality Index (SII) for DALYs (-53.17, 95% CI: -67.79, -38.55) highlights that low-SDI regions continue to bear a disproportionately high disease burden. From 1990 to 2021, health inequality in gastric cancer burden has widened, with lower-SDI countries failing to benefit equally from medical advancements and preventive efforts. This disparity underscores the urgent need for resource allocation, policy interventions, and investment in cancer care infrastructure in LMICs.

### Key risk factors: persistent behavioral and dietary risks

The decomposition analysis of attributable risk factors emphasizes that behavioral and dietary risks remain the predominant contributors to gastric cancer deaths, particularly in low-SDI regions:

1. Smoking, high-sodium diets, and low fruit and vegetable intake remain leading risk factors, contributing to sustained disease burden.
2. While smoking-related risks have declined globally, sodium-related risks remain prevalent, particularly in East Asia and Central Asia, highlighting the need for public health initiatives promoting dietary modifications and salt reduction policies.
3. Persistent H. pylori infection remains a major contributor in high-burden regions, necessitating comprehensive eradication programs and improved sanitation measures.

## Limitations

Several limitations of this study should be noted. First, as a GBD-based analysis, the estimates rely on secondary data sources and modeling assumptions, which may introduce measurement error or bias, particularly in low-SDI regions where cancer registry coverage is limited. Second, although temporal trends were examined using both EAPC and Joinpoint regression, the analysis was restricted to aggregated population-level indicators and could not account for tumor subtypes, histological variations, or treatment differences that may partly explain the observed heterogeneity across age groups and regions. Third, reproductive and hormonal factors, which are likely to play a key role in shaping gastric cancer risk among women of reproductive age, were not directly measured in the GBD dataset; therefore, relevant biological inferences should be interpreted cautiously and in conjunction with external epidemiologic or mechanistic evidence. Finally, projections to 2050 are inherently subject to uncertainty, as future changes in risk factors, healthcare systems, and prevention strategies may alter disease trajectories.

A significant limitation of our study is the inability to account for heterogeneity within the broad group of ‘women of reproductive age.’ The GBD dataset does not include individual-level information on reproductive history (e.g., parity, age at first birth), menopausal status, or use of exogenous hormones (e.g., oral contraceptives). These factors have been linked to gastric cancer risk in etiological research but could not be explored in our ecological analysis. Consequently, our study treats this demographic as a single group, and future studies that combine population-level trends with individual-level data from cohorts are warranted to disentangle these complex relationships.

### COVID-19 considerations

Estimates for 2020–2021 may have been influenced by pandemic-related changes in access to endoscopy, diagnostic delays, and registry operations. We retained these years to reflect the population’s actual experience, because these years capture real-world disruptions in screening, diagnosis, and cancer care delivery and thus preserve policy-relevant information. Trends spanning this period should be interpreted with appropriate caution.However, cross-period comparisons should be interpreted cautiously, and potential under- or over-ascertainment cannot be excluded.

### Sensitivity analyses

This work was unable to conduct formal sensitivity analyses (e.g., exclusion of pandemic years, alternative standard populations, or varying joinpoint caps) for this revision. Nevertheless, convergent patterns across methods, metrics, and stratifications—together with 95% uncertainty intervals—provide indirect reassurance that the main conclusions are not driven by a single modeling choice. Scenario-based sensitivity analyses will be prioritized in future work when feasible.

Projections may be influenced by unmeasured confounding (e.g., pandemic-related care disruptions, registry completeness, and health-system shocks), particularly in low-SDI regions. Estimates for 2050 in data-sparse settings should therefore be interpreted cautiously.

## Policy implications and recommendations

Given these findings, several policy recommendations are essential for further reducing the burden of gastric cancer among women of reproductive age:

1. Expand H. pylori screening and eradication programs, particularly in high-risk regions.
2. Enhance early detection strategies, including endoscopic screening initiatives in high-incidence areas.
3. Implement dietary interventions and public health campaigns, focusing on reducing sodium intake and increasing fruit and vegetable consumption.
4. Strengthen tobacco and alcohol control measures to mitigate behavioral risk factors.
5. Address healthcare access disparities in LMICs, ensuring equitable distribution of cancer prevention and treatment resources.

## Data Availability

The original contributions presented in the study are included in the article/**Supplementary Material**. Further inquiries can be directed to the corresponding author.
