# Supplementary material for: Global burden and future trends of gastric cancer in women of reproductive age: estimates from the GBD 2021 Study, 1990–2050
Source: Front Oncol. 2025 Nov 14;15:1616936. doi: 10.3389/fonc.2025.1616936 (PMC12661426; doi:10.3389/fonc.2025.1616936)
Supplement: Supplementary file 1 [file DataSheet1.zip › Appendix Figure A12.PDF]

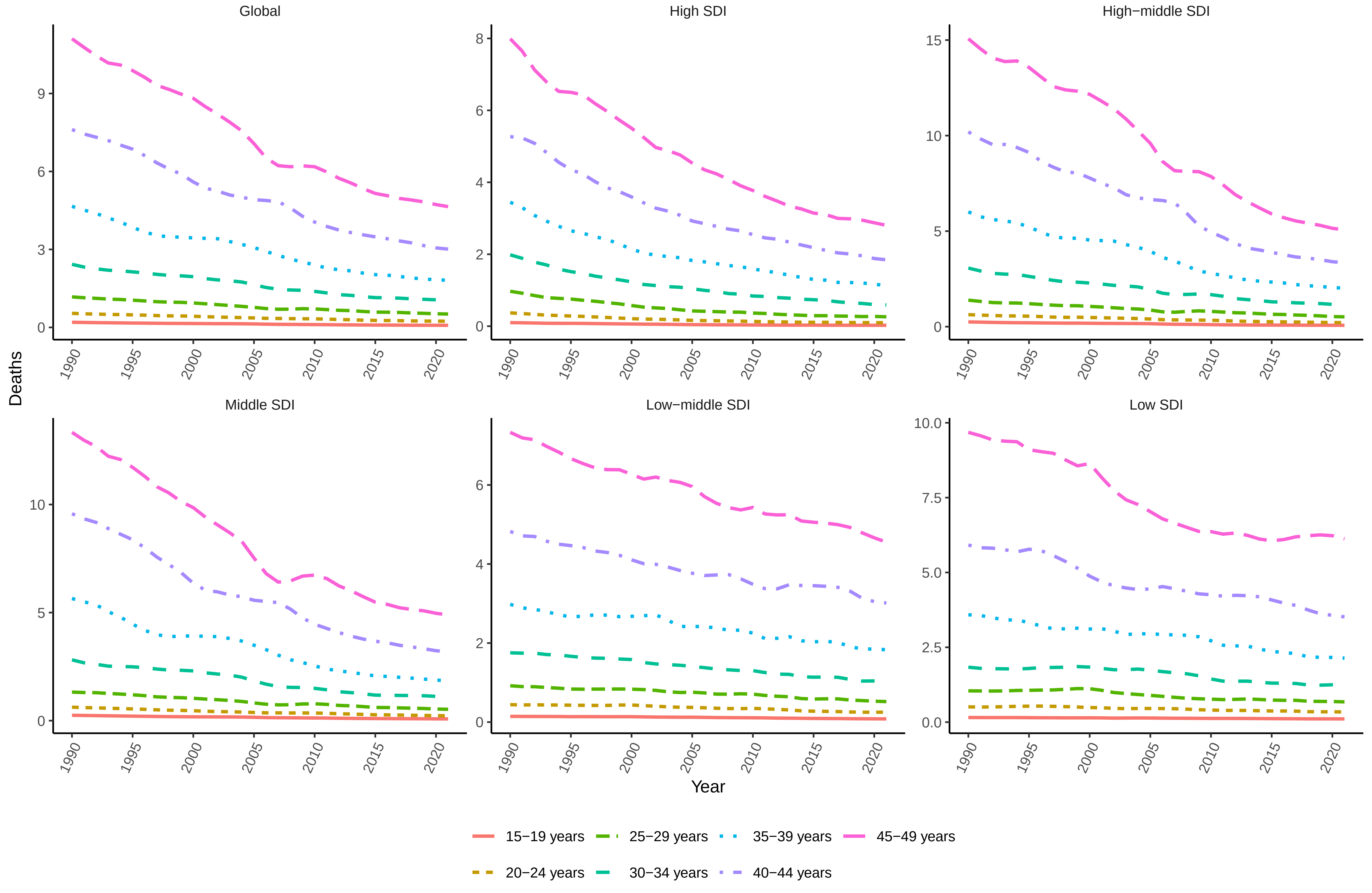

Global
High SDI
High−middle SDI
Middle SDI
Low−middle SDI
Low SDI
Deaths
Year
0
3
6
9
2
4
8
5
10
15
7.5
2.5
0.0
5.0
10.0
1990
1995
2000
2005
2010
2015
2020
15−19 years
20−24 years
25−29 years
30−34 years
35−39 years
40−44 years
45−49 years
