# Supplementary figures and images for: Global burden and future trends of gastric cancer in women of reproductive age: estimates from the GBD 2021 Study, 1990–2050
Source: Front Oncol. 2025 Nov 14;15:1616936. doi: 10.3389/fonc.2025.1616936 (PMC12661426; doi:10.3389/fonc.2025.1616936)

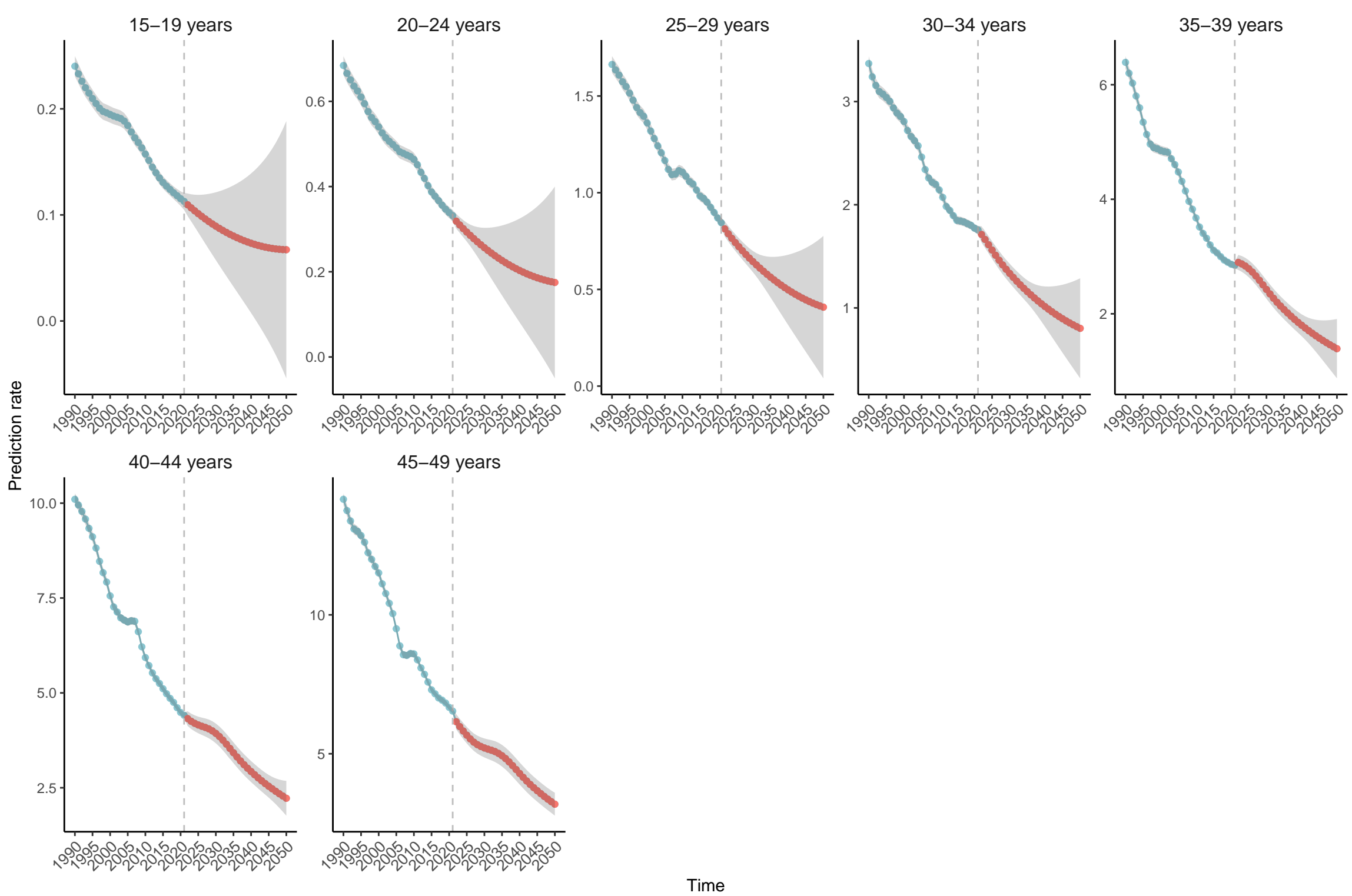

Supplement: Supplementary file 1 [file DataSheet1.zip › Appendix Figure A1.PDF]

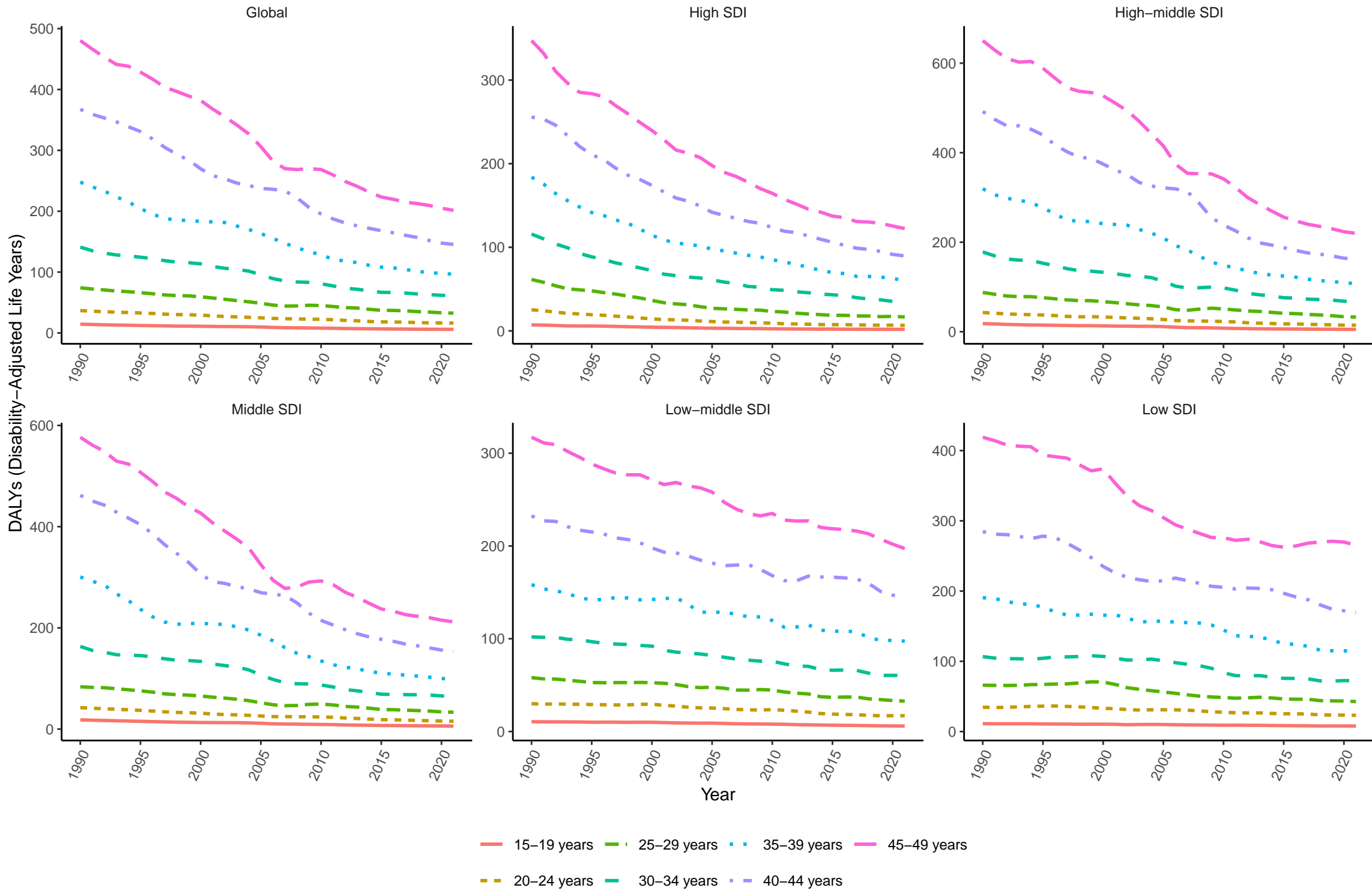

Supplement: Supplementary file 1 [file DataSheet1.zip › Appendix Figure A10.PDF]

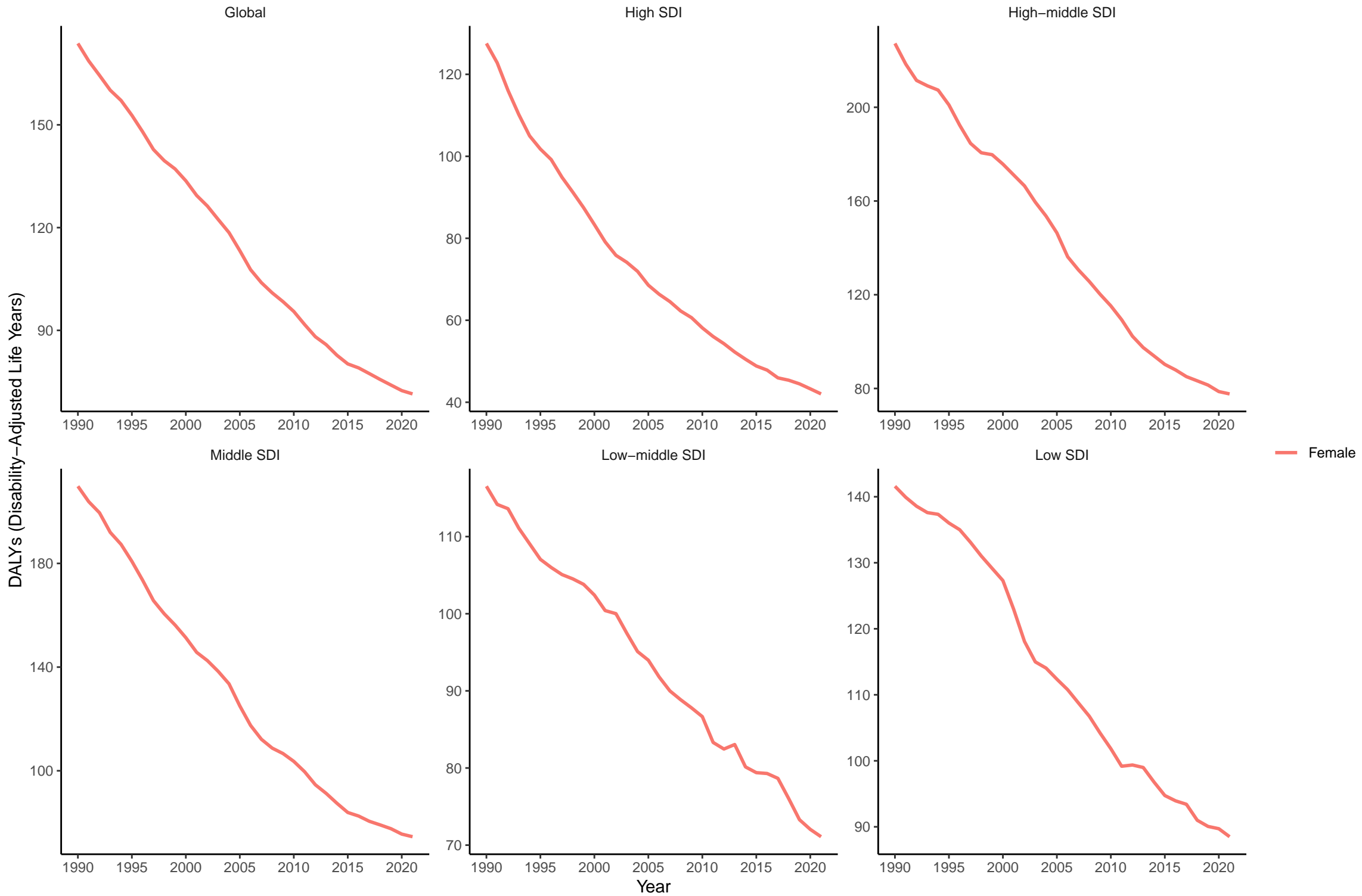

Supplement: Supplementary file 1 [file DataSheet1.zip › Appendix Figure A11.PDF]

Deaths

Global

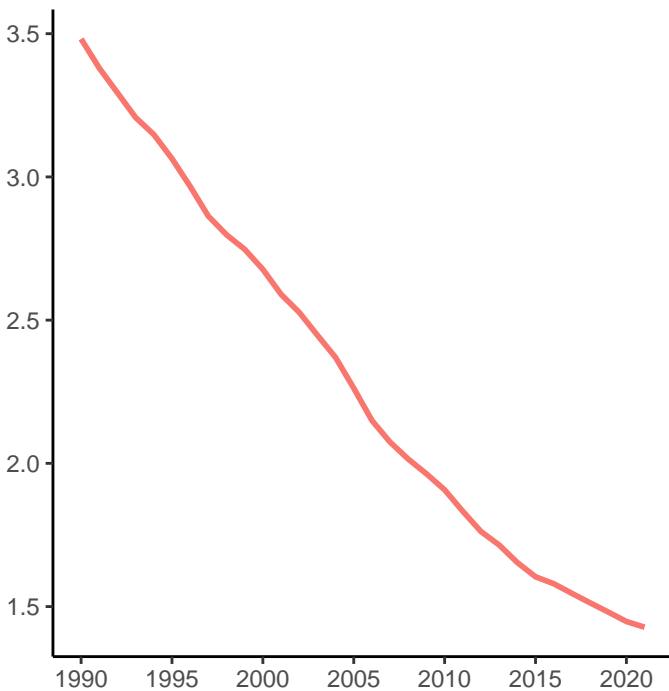

High SDI

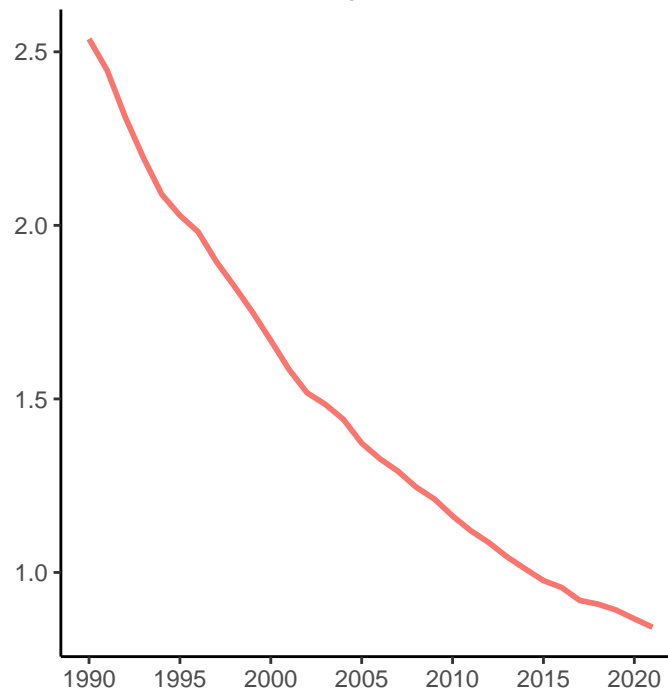

High-middle SDI

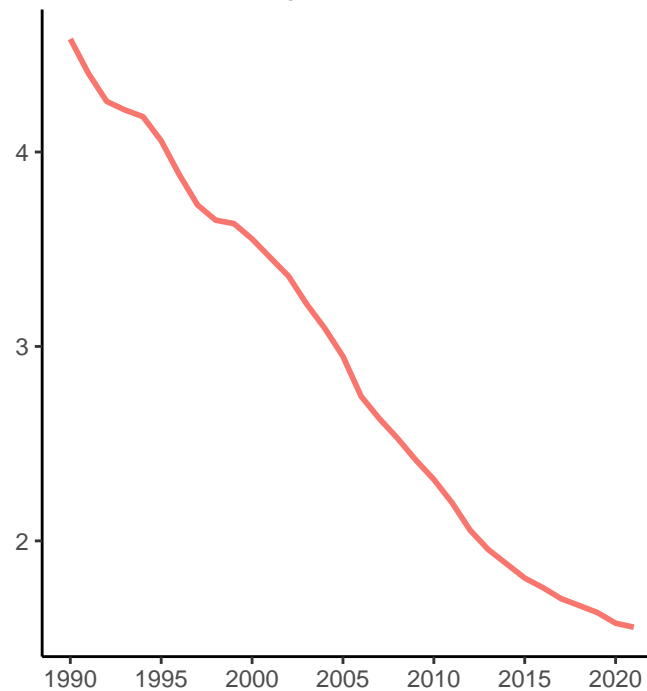

Middle SDI

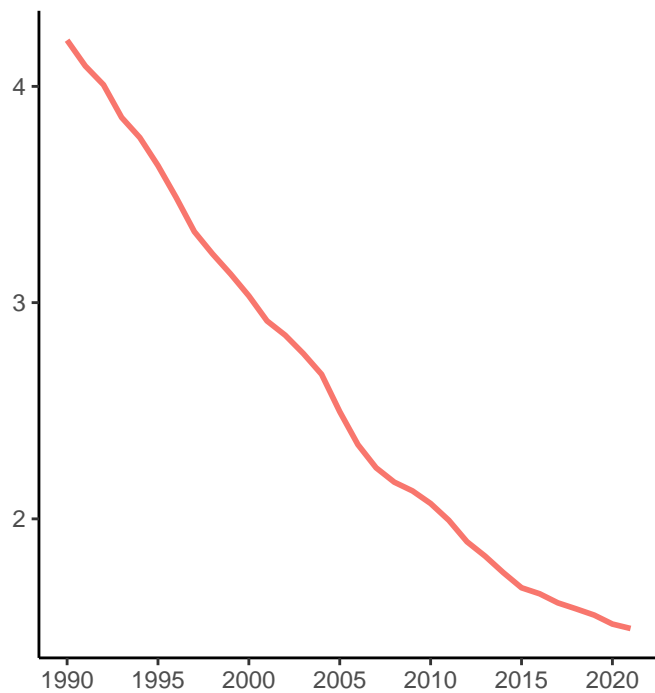

Low-middle SDI

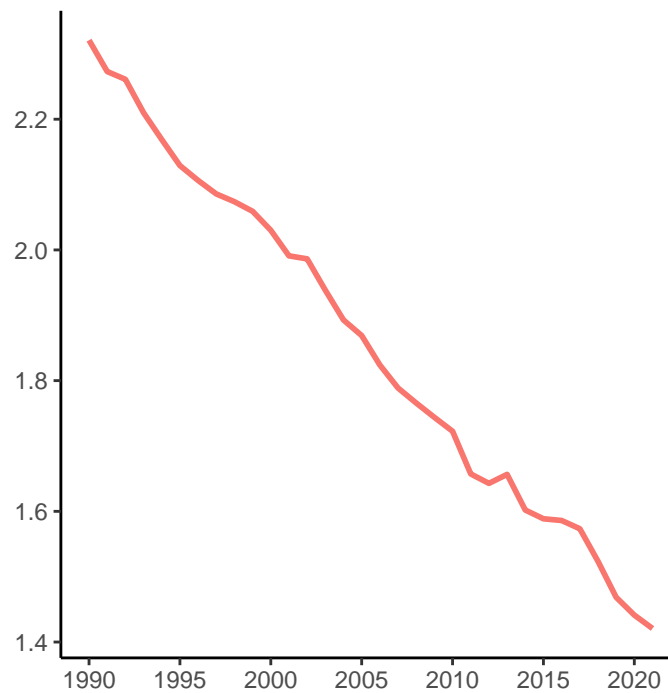

Low SDI

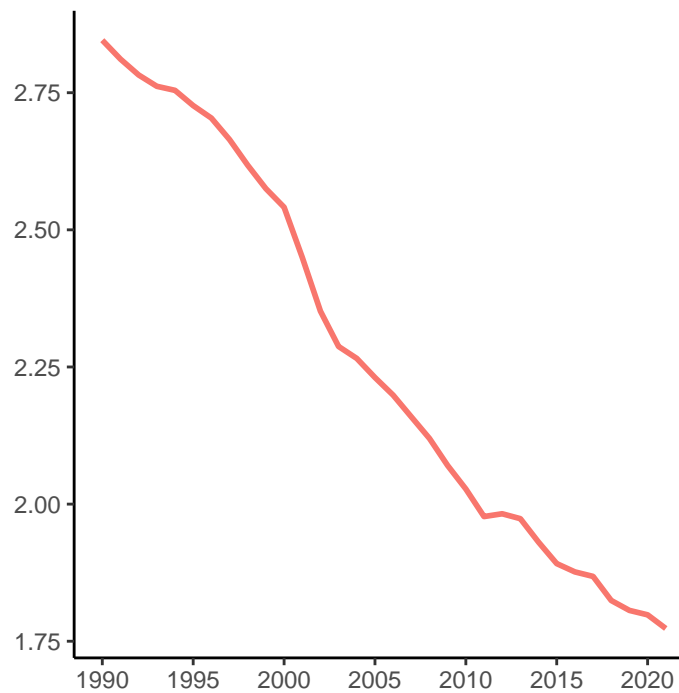

Female

Year

Supplement: Supplementary file 1 [file DataSheet1.zip › Appendix Figure A13.PDF]

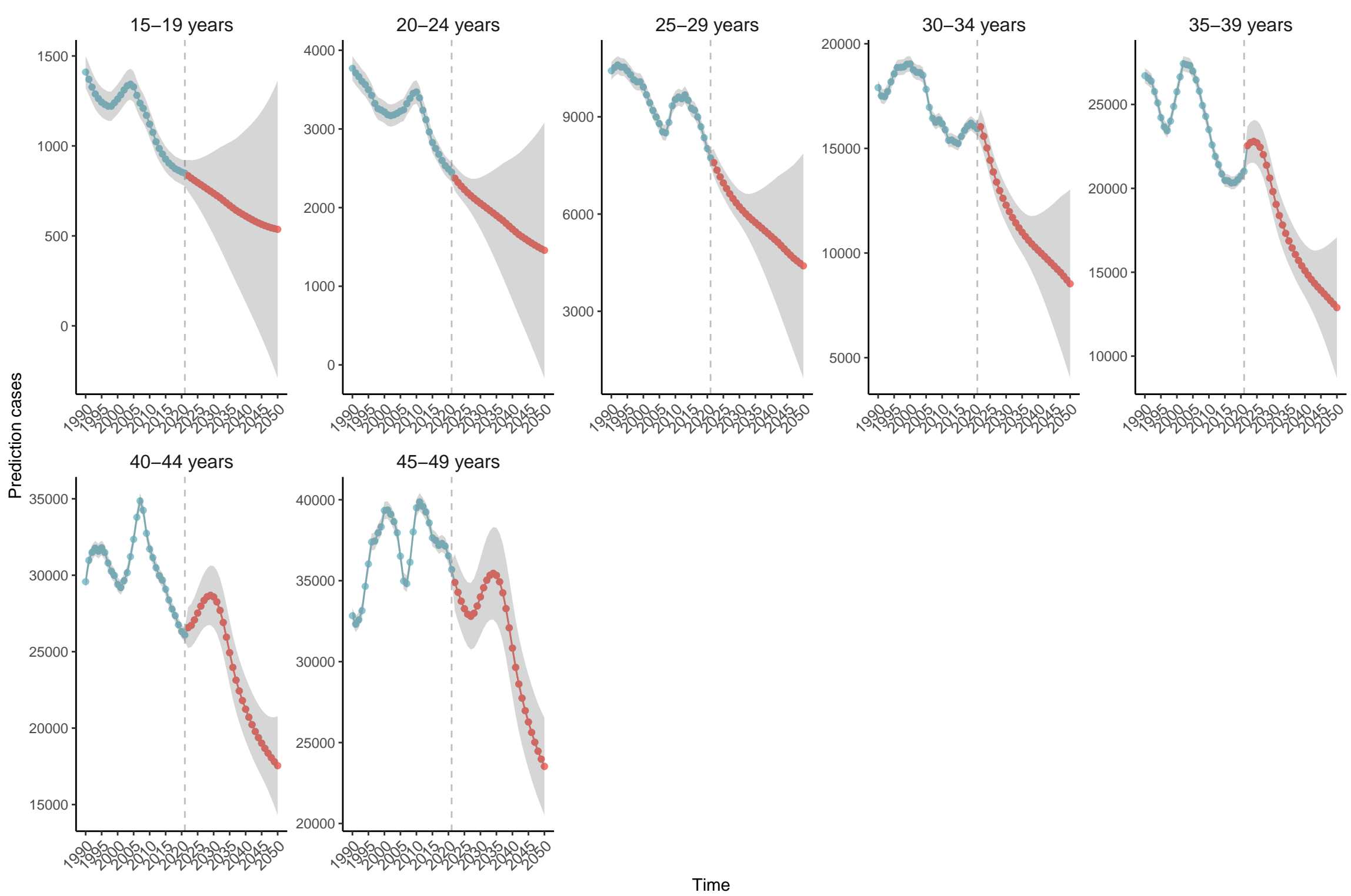

Supplement: Supplementary file 1 [file DataSheet1.zip › Appendix Figure A14.PDF]

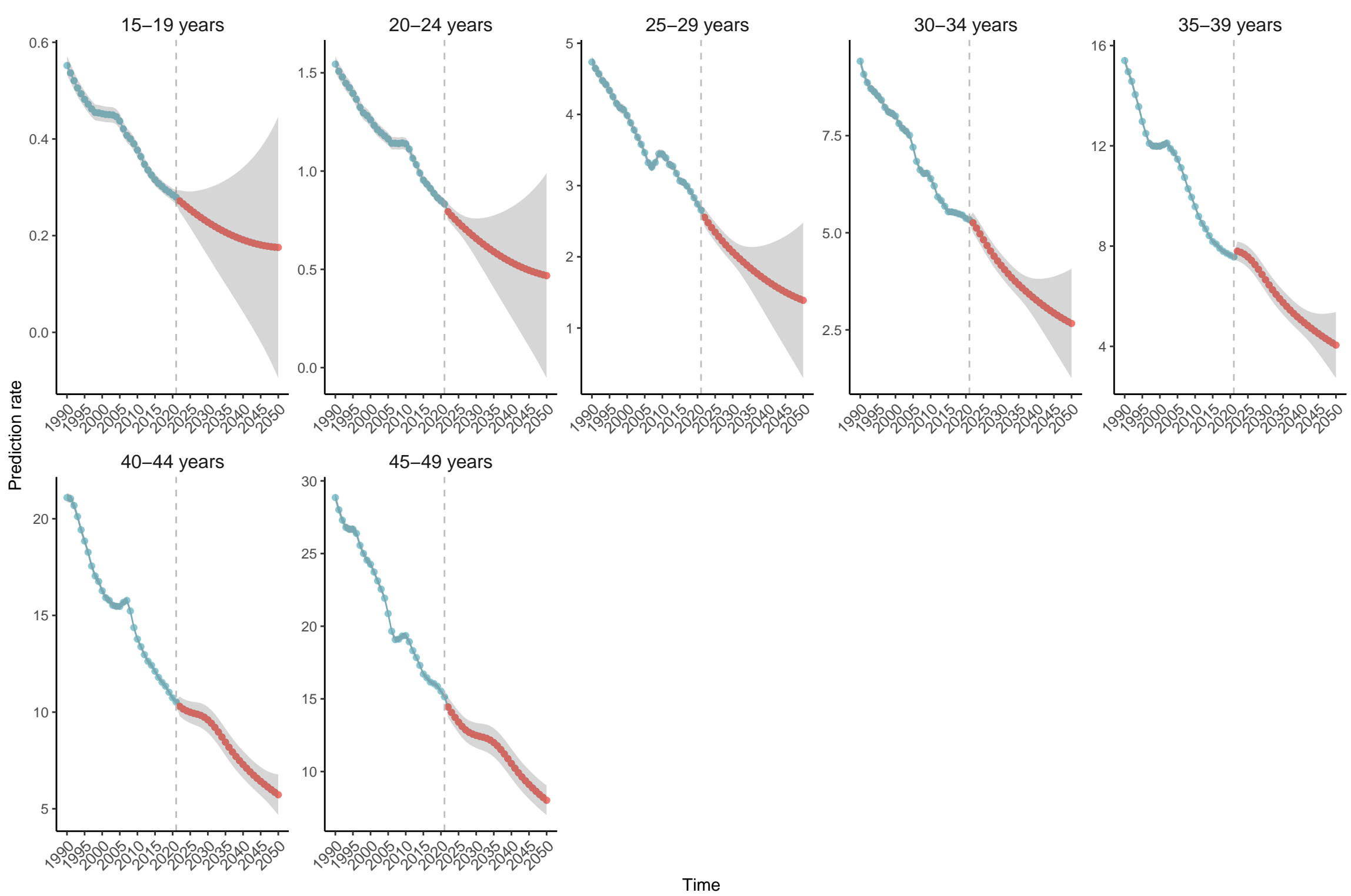

Supplement: Supplementary file 1 [file DataSheet1.zip › Appendix Figure A15.PDF]

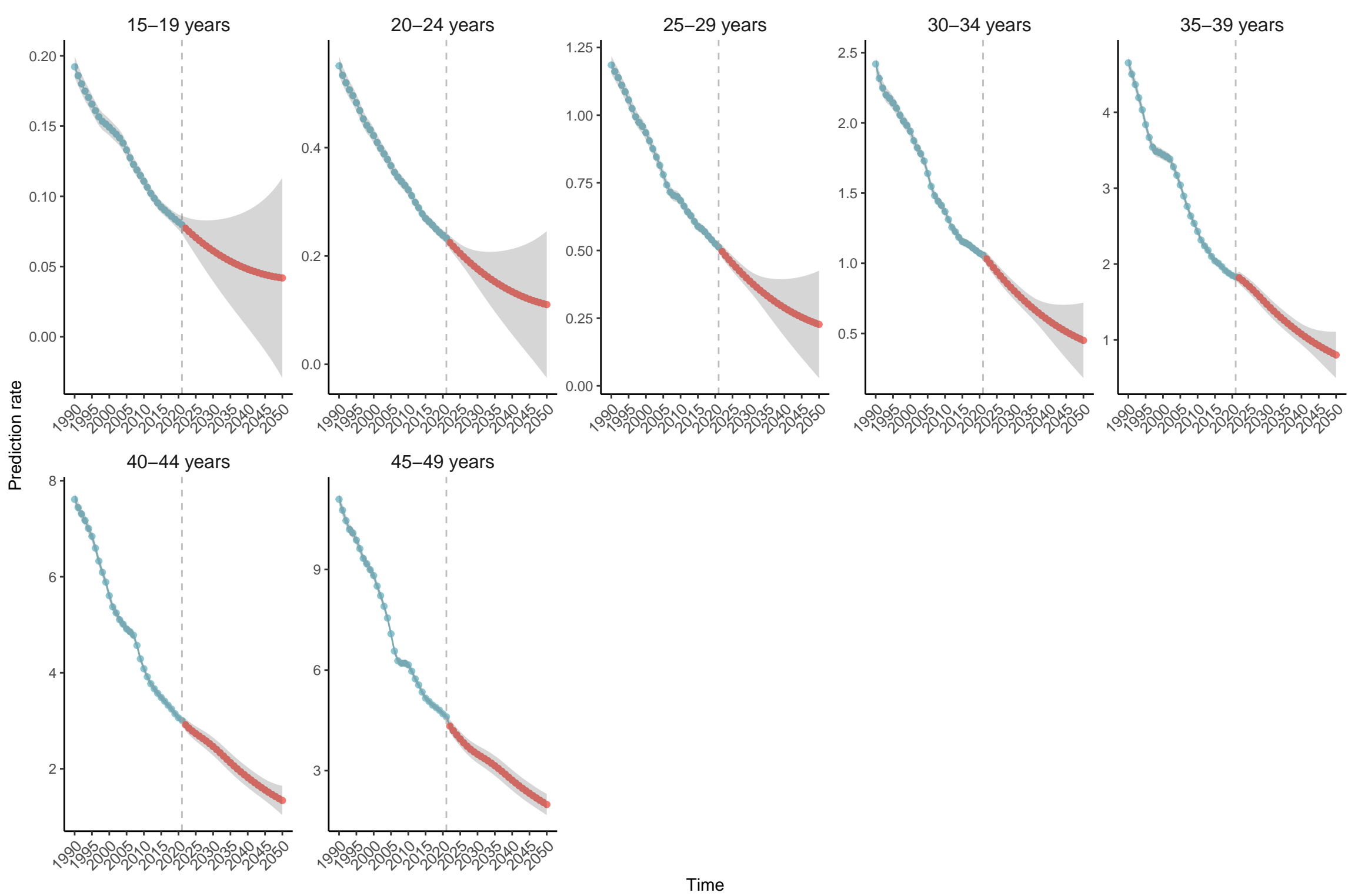

Supplement: Supplementary file 1 [file DataSheet1.zip › Appendix Figure A16.pdf]

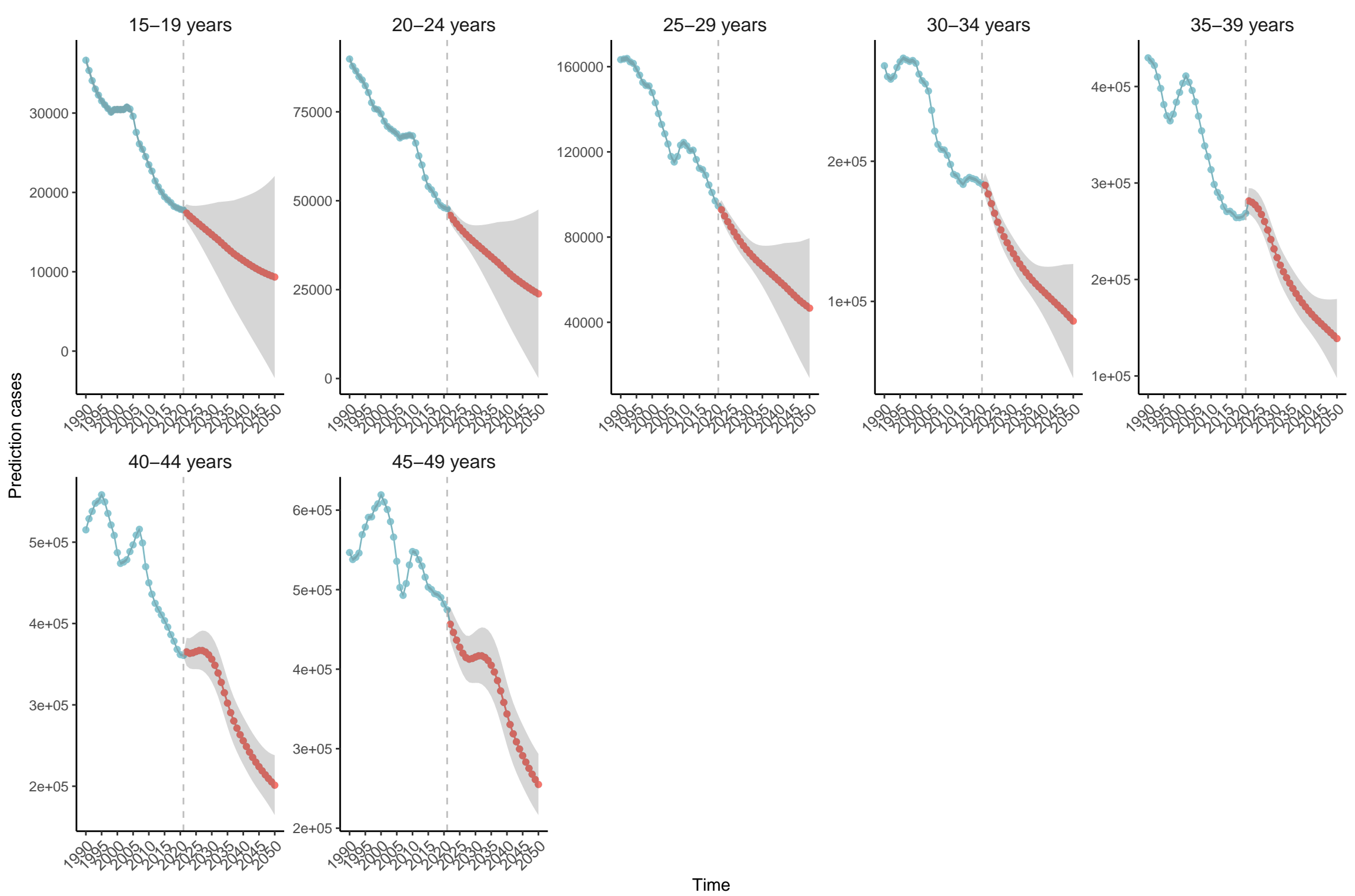

Supplement: Supplementary file 1 [file DataSheet1.zip › Appendix Figure A2.PDF]

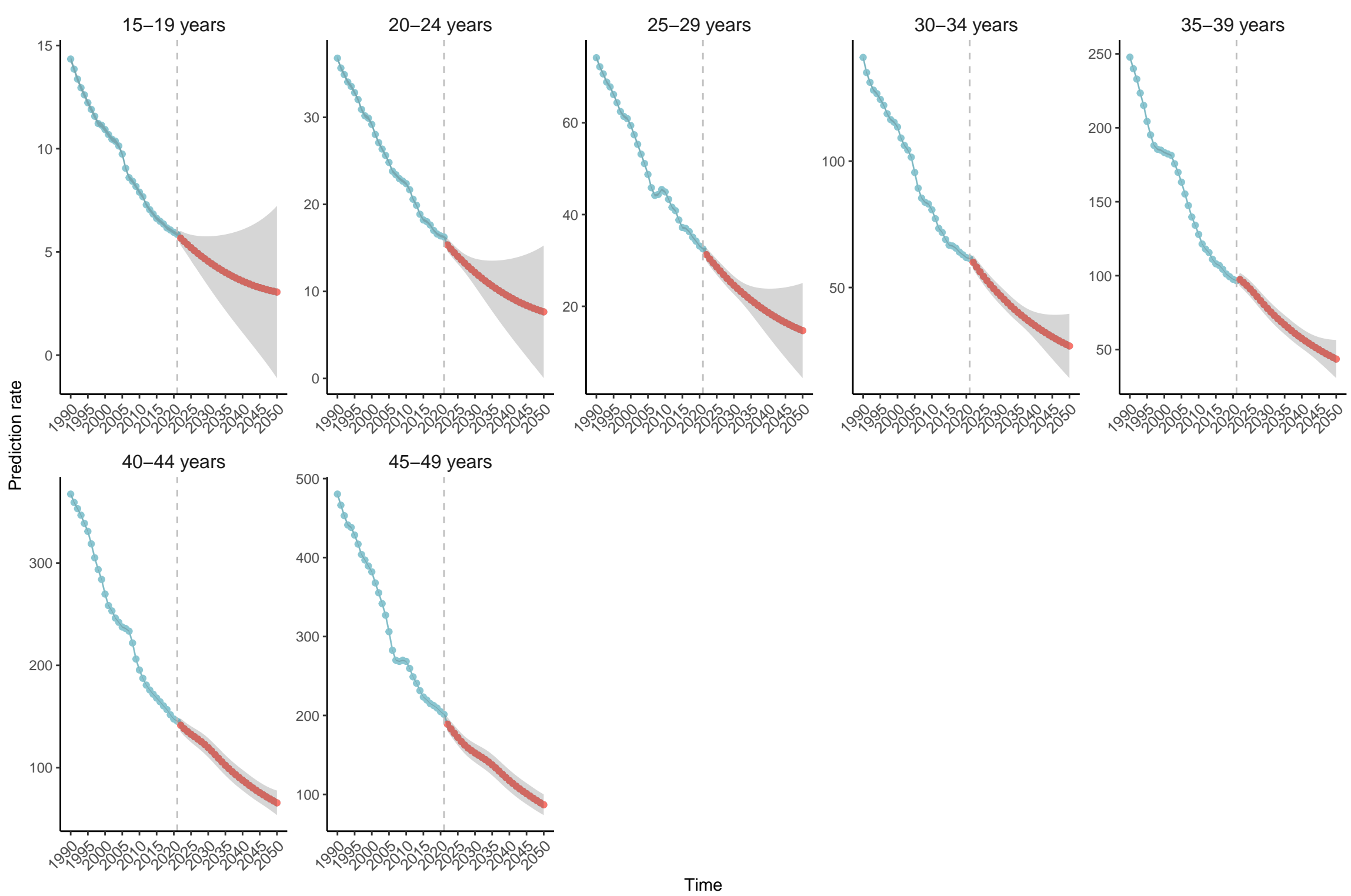

Supplement: Supplementary file 1 [file DataSheet1.zip › Appendix Figure A3.PDF]

Prediction cases

15–19 years

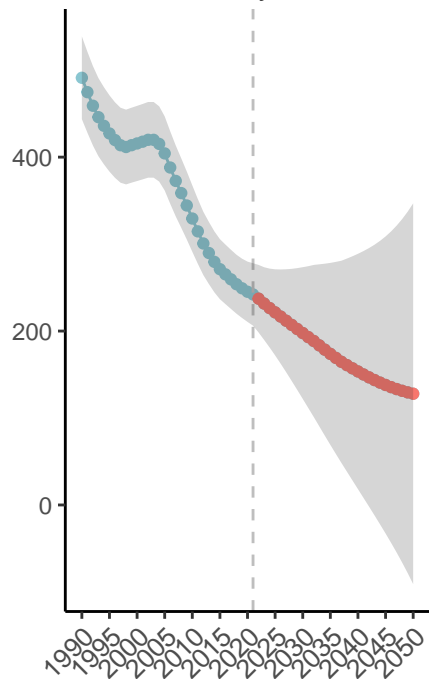

20–24 years

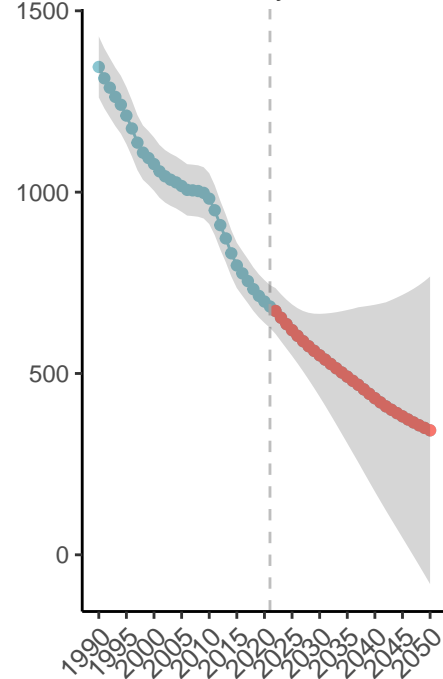

25–29 years

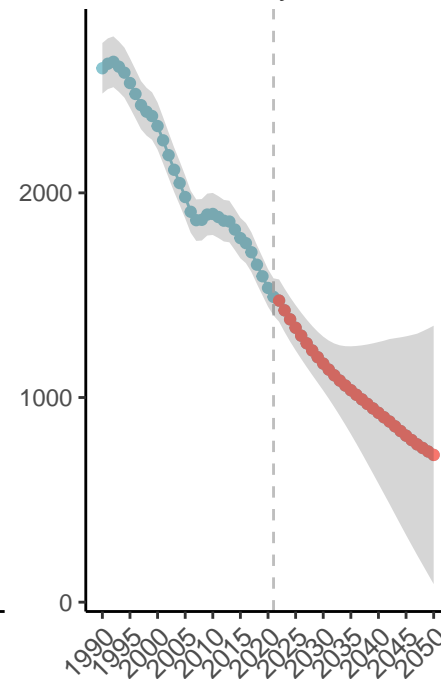

30–34 years

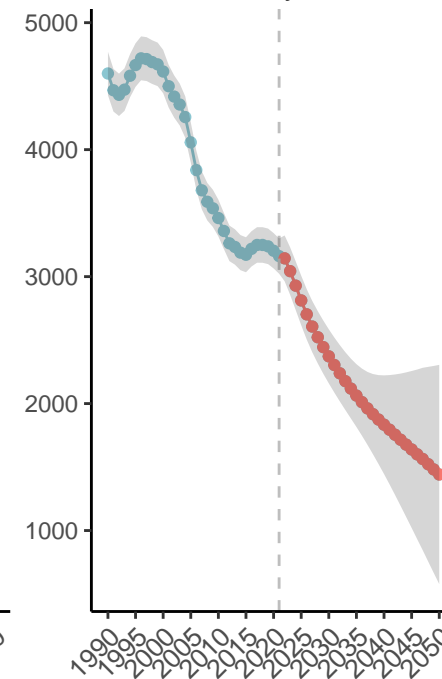

35–39 years

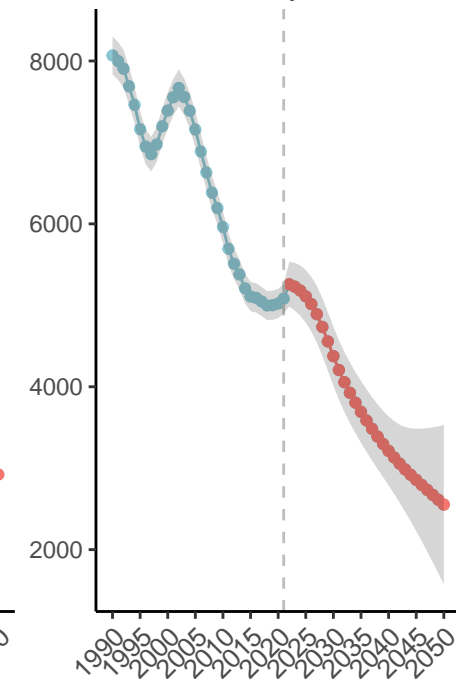

40–44 years

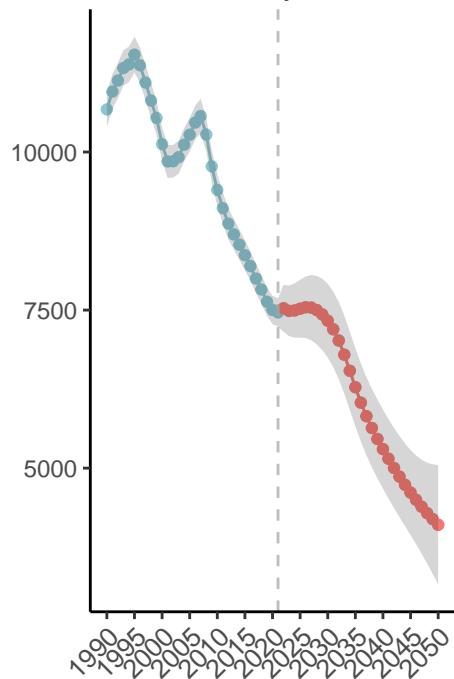

45–49 years

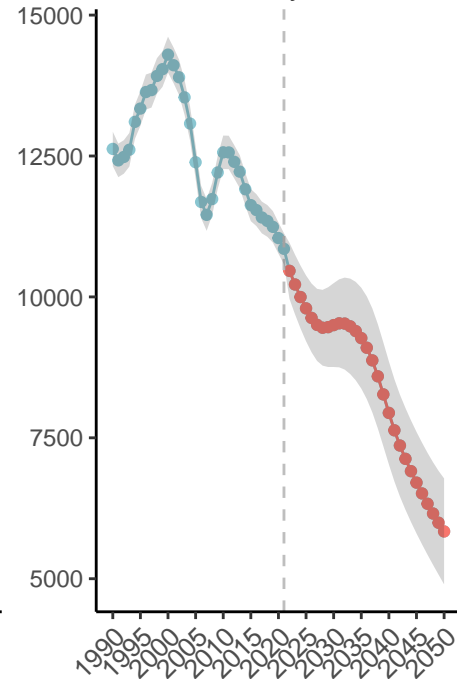

Time

Supplement: Supplementary file 1 [file DataSheet1.zip › Appendix Figure A4.PDF]

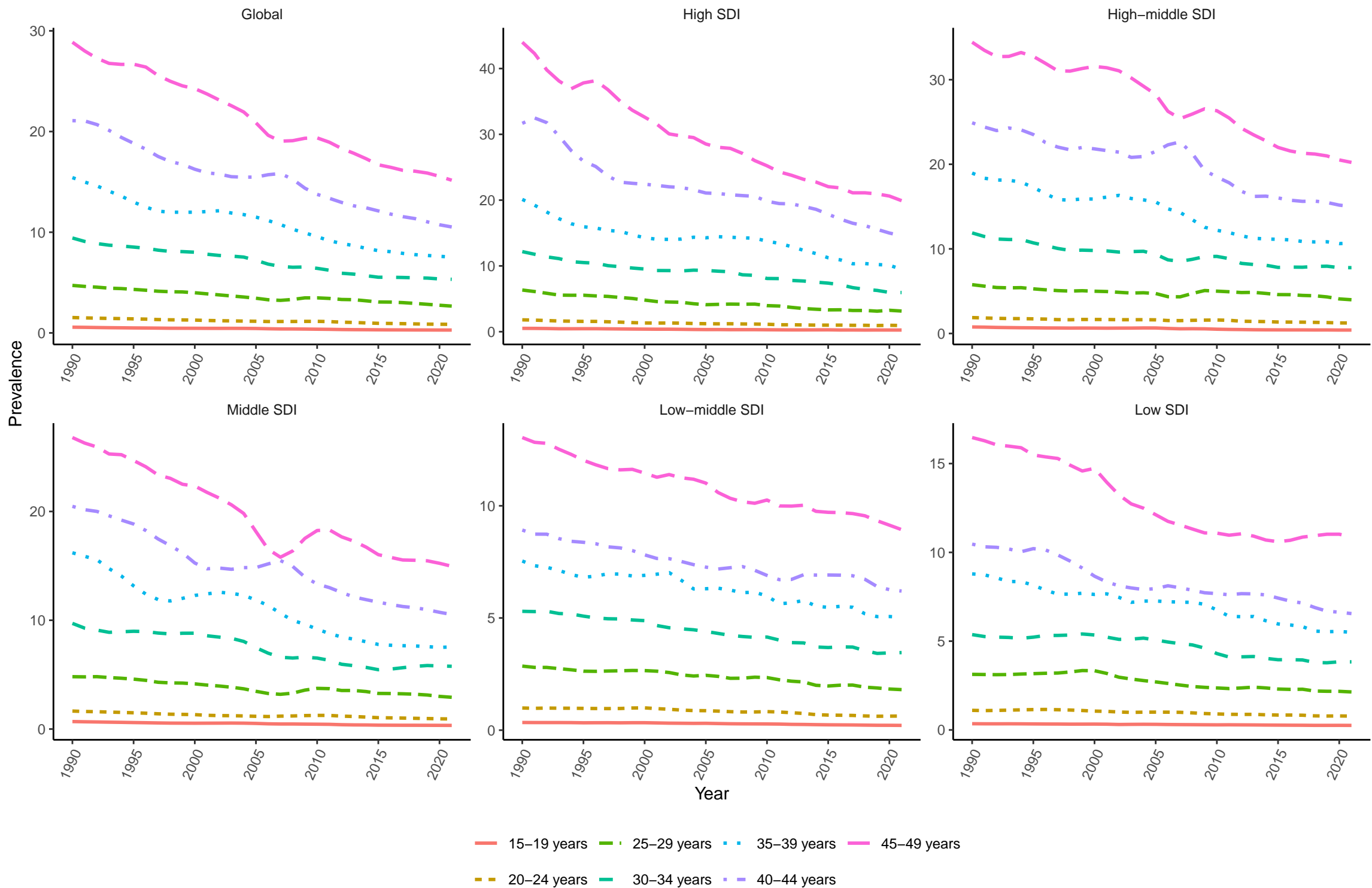

Supplement: Supplementary file 1 [file DataSheet1.zip › Appendix Figure A6.PDF]

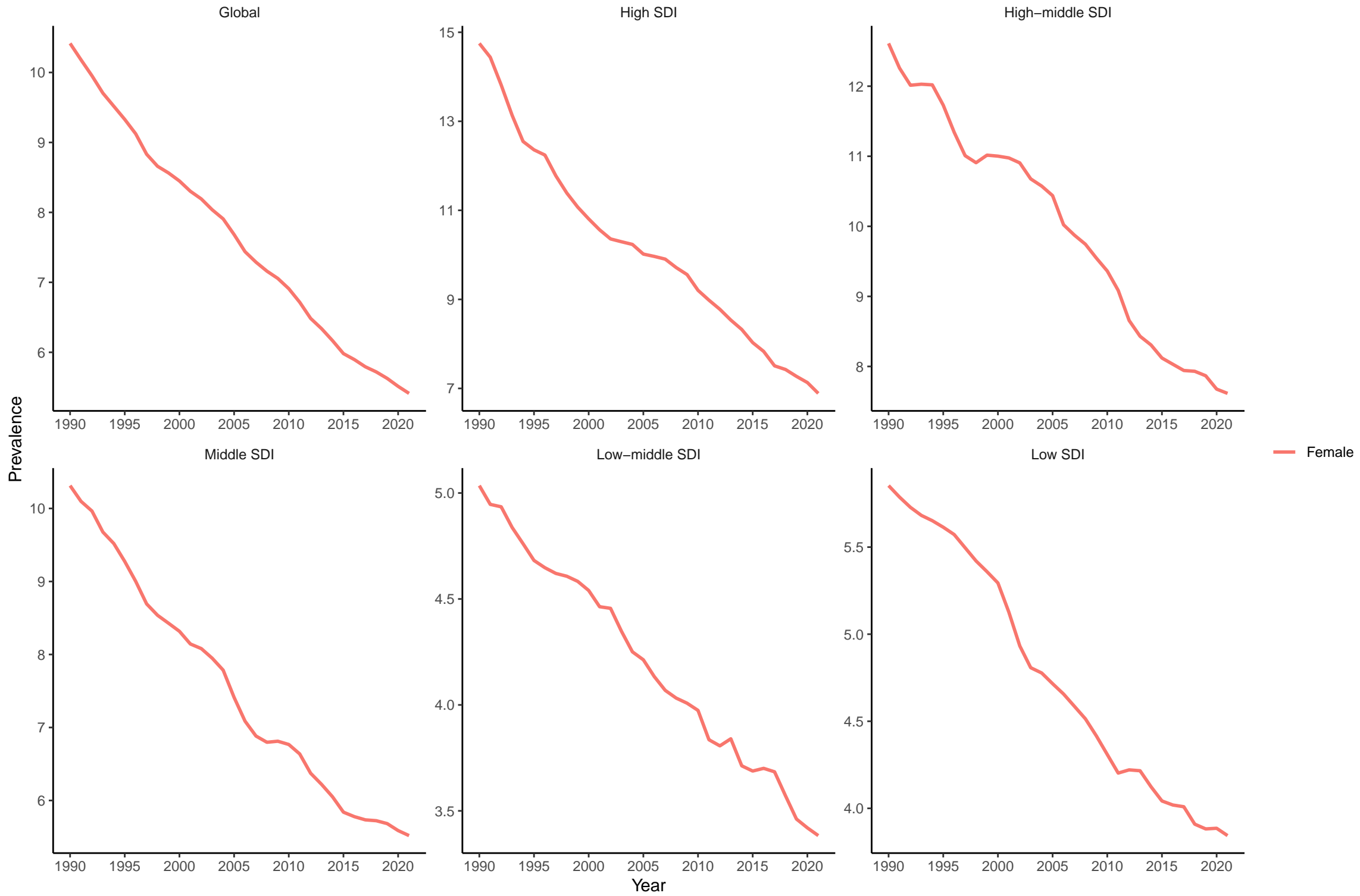

Supplement: Supplementary file 1 [file DataSheet1.zip › Appendix Figure A7.PDF]

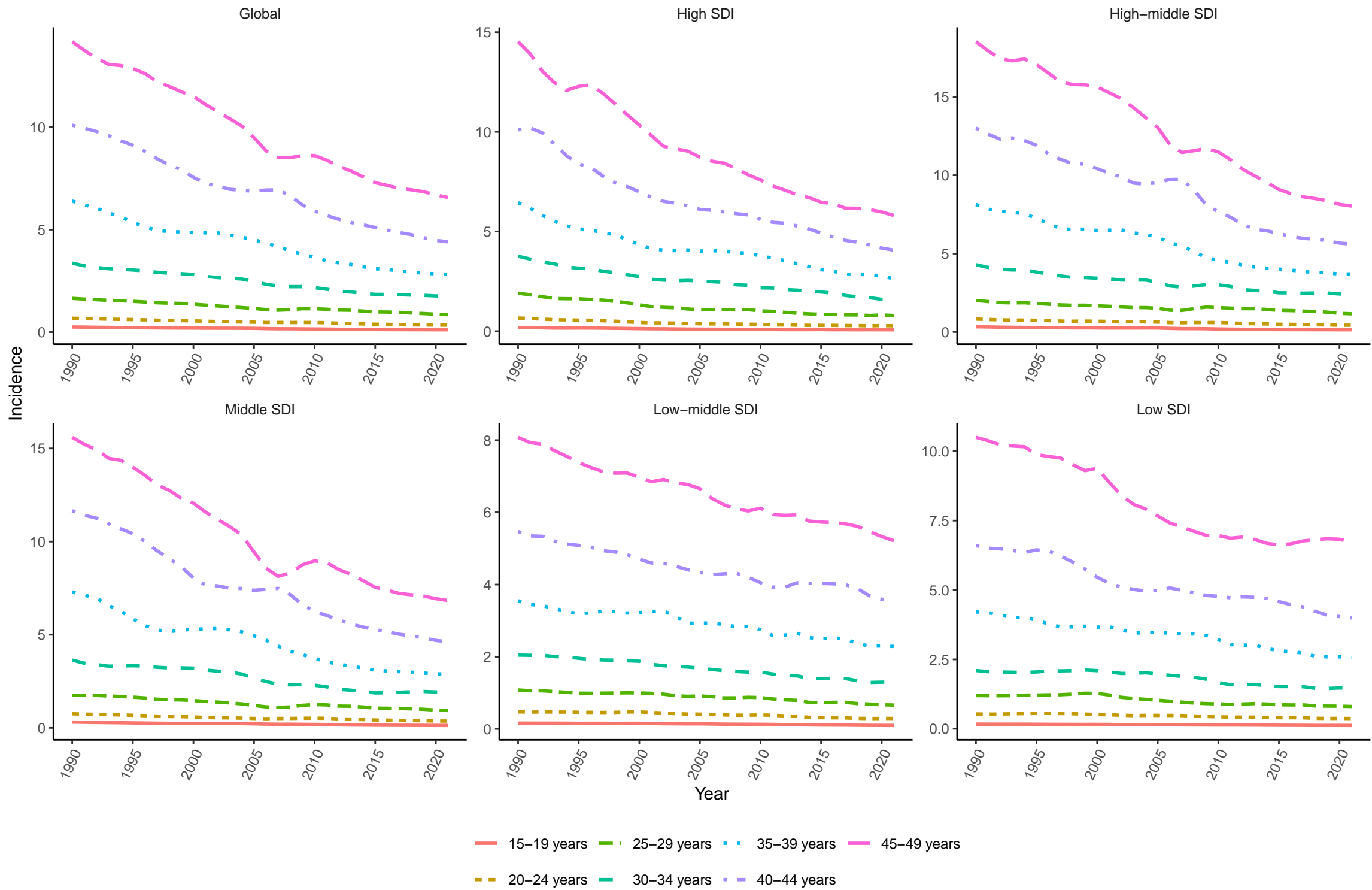

Supplement: Supplementary file 1 [file DataSheet1.zip › Appendix Figure A8.PDF]

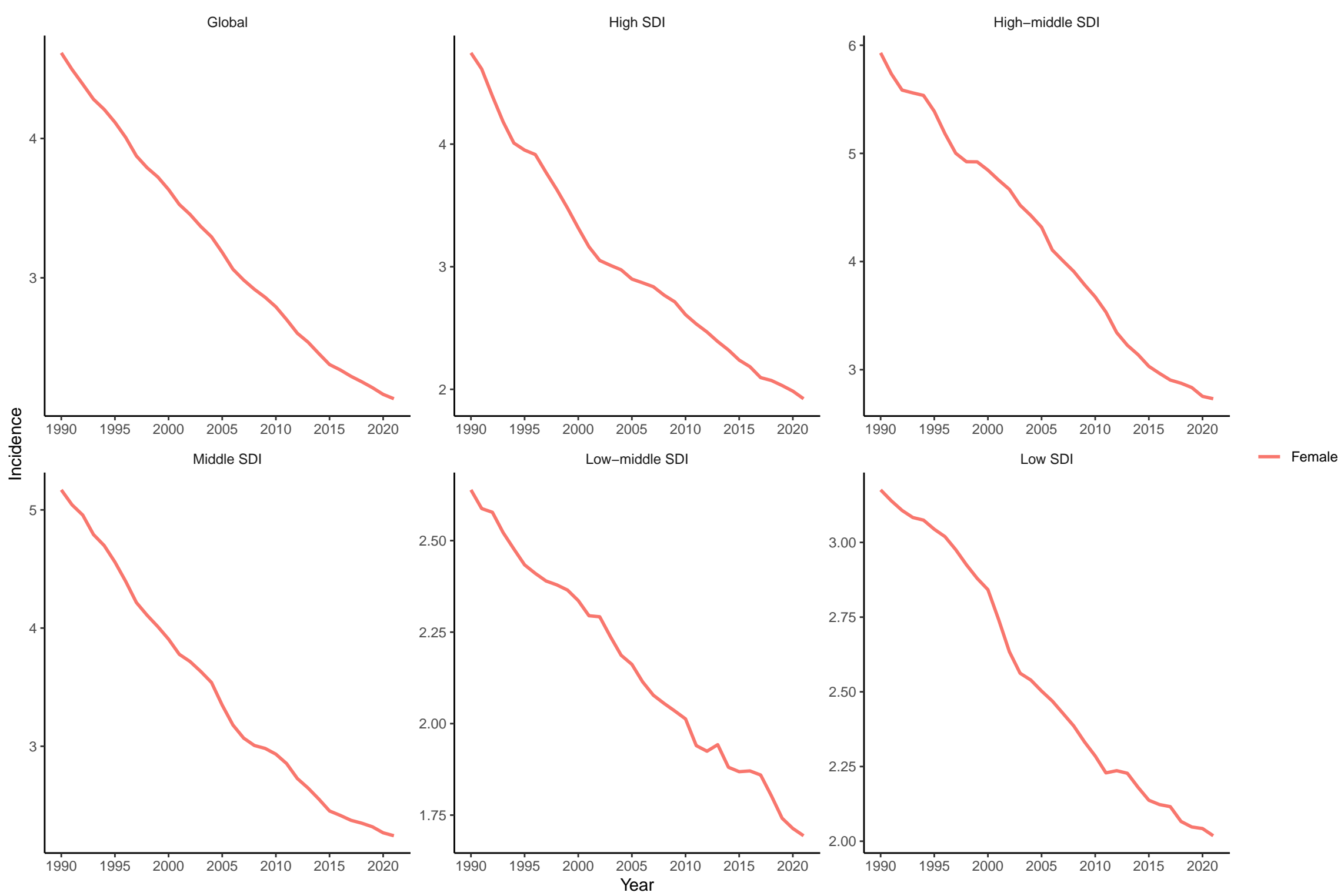

Supplement: Supplementary file 1 [file DataSheet1.zip › Appendix Figure A9.PDF]
